# Resources Required for Cervical Cancer Prevention in Low- and Middle-Income Countries

**DOI:** 10.1371/journal.pone.0164000

**Published:** 2016-10-06

**Authors:** Nicole G. Campos, Monisha Sharma, Andrew Clark, Jane J. Kim, Stephen C. Resch

**Affiliations:** 1 Center for Health Decision Science, Department of Health Policy and Management, Harvard T. H. Chan School of Public Health, 718 Huntington Avenue, Boston, Massachusetts, 02115, United States of America; 2 International Clinical Research Center, Department of Global Health, University of Washington, 908 Jefferson Street, Seattle, Washington, 98104, United States of America; 3 Department of Health Services Research and Policy, London School of Hygiene & Tropical Medicine, 15-17 Tavistock Place, London, WC1H9SH, United Kingdom; Universidade Estadual de Maringa, BRAZIL

## Abstract

**Background:**

Cervical cancer is the fourth leading cause of cancer death in women, with 85% of cases and deaths occurring in developing countries. While organized screening programs have reduced cervical cancer incidence in high-income countries through detection and treatment of precancerous lesions, the implementation of organized screening has not been effective in low-resource settings due to lack of infrastructure and limited budgets. Our objective was to estimate the cost of comprehensive primary and secondary cervical cancer prevention in low- and middle-income countries.

**Methods and Findings:**

We performed a modeling analysis to estimate 1) for girls aged 10 years, the cost of 2-dose human papillomavirus (HPV) vaccination; and 2) for women aged 30 to 49 years, the cost of cervical cancer screening (with visual inspection with acetic acid (VIA), HPV testing, or cytology) and preventive treatment in 102 low- and middle-income countries from 2015 to 2024. We used an Excel-based costing and service utilization model to estimate financial costs (2013 US$) based on prevalence of HPV, prevalence of precancerous lesions, and screening test performance. Where epidemiologic data were unavailable, we extrapolated from settings with data using an individual-based microsimulation model of cervical carcinogenesis (calibrated to 20 settings) and multivariate regression. Total HPV vaccination costs ranged from US$8.6 billion to US$24.2 billion for all scenarios considered (immediate, 5-year, or 10-year roll-out; price per dose US$4.55-US$70 by country income level). The total cost of screening and preventive treatment ranged from US$5.1 billion (10-year roll-out, screening once at age 35 years) to US$42.3 billion (immediate roll-out, high intensity screening). Limitations of this analysis include the assumption of standardized protocols by country income level that did not account for the potential presence of multiple screening modalities or management strategies within a country, and extrapolation of cost and epidemiologic data to settings where data were limited.

**Conclusions:**

The estimated cost of comprehensive cervical cancer prevention with 2-dose HPV vaccination of 10-year-old girls and screening of women aged 30 to 49 years ranges from US$13.7 billion to US$66.5 billion, depending on speed of roll-out, vaccine price per dose, and screening test and frequency. Findings demonstrate the substantial impact of vaccine price in middle-income countries that are not eligible for assistance from Gavi, the Vaccine Alliance. Replacing routine cytology with HPV-based screening may reduce total costs. Data on the health impact and relative cost-effectiveness of strategies are needed to determine the best value for public health dollars.

## Introduction

Cervical cancer is the fourth most common cancer in women, resulting in an estimated 528,000 incident cases and 266,000 deaths worldwide in 2012 [1]. While organized screening programs have reduced cervical cancer incidence in high-income countries through detection and treatment of precancerous lesions, the implementation of organized screening has not been effective in low-resource settings due to the lack of health delivery infrastructure and limited financial resources. Approximately 85% of cases and deaths occur in low- and middle-income countries (LMIC) [1], often affecting young women who are critical to social and economic stability.

New opportunities to reduce preventable deaths from cervical cancer stem from two human papillomavirus (HPV) vaccines—both with high efficacy against HPV types 16 and 18 (HPV-16/18), which cause approximately 70% of cervical cancers—and point-of-care HPV-based testing designed for low-resource settings [2–4]. In 2013, Gavi, the Vaccine Alliance began providing support for HPV-16/18 vaccines to eligible countries to increase access to vaccination where the disease burden and financial need are greatest [5]. Screening with HPV testing and visual inspection with acetic acid (VIA) have been demonstrated to be effective [6–8] and potentially cost-effective [9] in low-resource settings, allowing for fewer follow-up visits (e.g., screen-and-treat approaches) and, in the case of HPV testing, automated processing of laboratory specimens that reduces resource and quality control requirements. Moreover, the World Health Organization has recently recommended the use of HPV testing or VIA for cervical cancer screening in those regions and countries that have not already established an effective, high-coverage Pap-based program [10].

In 2015, there were nearly 50 million 10-year-old girls and 760 million women of screening age in LMIC. To design and coordinate HPV vaccination and cervical cancer screening programs, decision makers must consider many attributes and outcomes associated with available prevention strategies, including: 1) feasibility, related to human resources, infrastructure, and financial capacity; 2) the likelihood of acceptability and political support; 3) health and economic impact; and 4) short- and long-term affordability.

Motivated by the need for information on financial cost requirements by those making immunization and screening policy recommendations—including the World Health Organization (WHO), financing coordination mechanisms (e.g., Gavi, the Vaccine Alliance; the Pan American Health Organization [PAHO] Revolving Fund), and potential donors—our objective was to estimate the financial cost of scaling up coverage of primary and secondary cervical cancer prevention for the total target population of women in LMIC from 2015 to 2024.

## Methods

### Analytic overview

We used a model-based approach to synthesize population and demographic data from 102 low- and middle-income countries, coupled with country-specific epidemiologic data. The Excel-based CERVIVAC model was used to project the costs of HPV-16/18 vaccination of pre-adolescent girls aged 10 years and screening of adult women aged 30 to 49 years, by income tier and World Bank region, under various scenarios of years to roll-out, vaccine price per dose, screening test, and screening frequency. Because epidemiologic data on the prevalence of HPV and precancerous lesions are not available in all countries, we estimated these inputs using multivariate regression models and extrapolating from an individual-based microsimulation model that was previously calibrated to 20 of the LMIC of interest.

This analysis was conducted from a payer perspective, including all direct medical costs (including but not limited to the price of the vaccine and screening tests) for pre-adolescent HPV vaccination, screening, and relevant diagnosis and treatment of precancerous lesions. We present both undiscounted costs and future costs discounted at an annual rate of 3% in 2013 US dollars (US$).

### CERVIVAC Model

The CERVIVAC model was developed for PAHO’s ProVac Initiative as a tool to enable Latin America and Caribbean country teams to conduct local cost-effectiveness analysis of cervical cancer prevention. CERVIVAC contains separate modules for evaluating the costs associated with vaccination and screening and treatment. The model, programmed using Microsoft Excel and Visual Basic for Applications 2007 (Microsoft Corporation, Redmond, WA), tracks multiple female birth cohorts starting at a target age (e.g., 10 years for HPV vaccination; 30 years for screening), projecting cost outcomes associated with HPV vaccination and screening and treatment by counting events that involve resource utilization and multiplying these events by a country-specific unit cost.

The HPV vaccination module counts the cost per dose and vaccine delivery costs, while the screening module counts the costs of screening visits, follow-up visits in triage strategies, cryotherapy, diagnostic confirmation with colposcopy for cytology (i.e., Pap)-based strategies and for women who are not eligible for screen-and-treat cryotherapy, and loop electrosurgical excision procedures (LEEP). Resource utilization associated with screening is driven by screening test characteristics (i.e., sensitivity and specificity), HPV prevalence, and the prevalence of precancerous lesions. We did not include costs associated with treatment of cervical cancer, as our objective was to estimate costs associated with prevention.

We included LMIC with population size greater than 1 million persons. We excluded countries that were missing basic data (e.g., United Nations population data, gross national income [GNI] per capita, WHO CHOICE facility visit cost estimates). The comprehensive list of 102 countries, stratified by income tier according to GNI per capita (Atlas method, 2013 US$),[11] is shown in **Table A in** S1 File; Lower Middle Income (LMI) and Upper Middle Income (UMI) countries have been further stratified at the midpoint GNI per capita into LMI1 and LMI2 and UMI1 and UMI2, respectively. Country stratification by world region is displayed in **Table B in** S1 File.

### Population and epidemiologic input data

For each country, demographic estimates were from the United Nations World Population Prospects 2012 data [12]. We estimated the number of females alive in each of the 102 countries, in each single year of age, and in each calendar year from 2015 to 2024. Each cohort was then tracked to capture relevant health service utilization associated with either HPV vaccination of 10-year old girls or screening of women aged 30 to 49 years from 2015 to 2024. As such, no scenarios involved combining both vaccination and screening within the same birth cohort.

To estimate HPV prevalence in countries without epidemiologic survey data, we constructed multivariate regression models using previously published methods[13]. We included the following variables in the regression models to predict HPV prevalence in countries with available data: country income classification (low, lower middle, upper middle) [11]; geographic region (Central and South America, Eastern Europe, Asia, North Africa, and sub-Saharan Africa) [11]; and age-specific cervical cancer incidence in ten-year age groups (age 25 to 34 years; age 35 to 44 years; age 45 to 54 years, age 55 to 64 years) from registry data when available (N = 99) [14]; else from Globocan 2012 (N = 16) [1]. The model was restricted to LMIC to control for the impact of screening. Generalized linear modeling for proportions with binomial family and log link was employed to assess the relationship between HPV prevalence and predictor variables. Four models were created (for women age 30 to 34 years; 35 to 39 years; 40 to 44 years; and 45 to 49 years). The models were then used to predict age-specific HPV prevalence for countries without prevalence survey data. Models were examined for goodness of fit, leverage, and normality. We describe adjustments for outliers in the S1 File; HPV prevalence inputs are presented in **Table C in** S1 File.

To estimate prevalence of precancerous lesions, we utilized an existing microsimulation model of cervical carcinogenesis to discern the typical relationship (i.e., age-specific ratio) between 1) cervical intraepithelial neoplasia grade 1 (CIN1) prevalence and oncogenic HPV prevalence; and 2) cervical intraepithelial neoplasia grade 2 or 3 (CIN2/3) prevalence and detected cancer incidence in the absence of screening and treatment. We selected these relationships given the association of CIN1 and CIN2/3 with HPV infection and cervical cancer incidence, respectively, for which we have empirical data in many countries. The previously described microsimulation model has been calibrated to epidemiologic data from the following low- and middle-income settings: Argentina, Brazil, China, Colombia, Costa Rica, Haiti, India, Kenya, Lebanon, Mexico, Mozambique, Nigeria, Peru, South Africa, Tanzania, Thailand, Turkey, Uganda, Vietnam, Zimbabwe [15–22].

To determine the relationship between prevalence of CIN1 and HPV prevalence, we used microsimulation model output from each of the 20 calibrated country models to determine the median age-specific ratio of CIN1 prevalence to oncogenic HPV prevalence for women aged 30 to 49 years. We applied this ratio to actual or predicted age-specific HPV prevalence (**Table C in** S1 File) to estimate age-specific CIN1 prevalence in each country in the analysis. The ratios applied to HPV prevalence for each age group were as follows: 0.36 (age 30 to 34); 0.39 (age 35 to 39); 0.41 (age 40 to 44); and 0.41 (age 45 to 49).

To determine the relationship between CIN2/3 prevalence and cancer incidence, we used microsimulation model output from each of the 20 calibrated country models to determine the median age-specific ratio of CIN2/3 prevalence to detected cancer incidence for women aged 30 to 49 years. We applied this ratio to age-specific cancer incidence in 5-year intervals from Globocan 2012 [1] (**Table D in** S1 File) to estimate age-specific CIN2/3 prevalence in each country in the analysis. The ratios applied to cancer incidence rate per woman for each age group were as follows: 85.9 (age 30 to 34); 49.3 (age 35 to 39); 29.8 (age 40 to 44); and 22.0 (age 45 to 49). Applying these ratios to Globocan 2012 cancer incidence rates led to CIN2/3 prevalence estimates of 1–2% on average across countries, consistent with estimates reported in the literature [4,8,23–28].

### Vaccination and screening scenarios

We assumed 2-dose HPV vaccination, as recommended by the WHO [29], of girls aged 10 years. Screening scenarios were based on country income classification and WHO guidelines [10]. Seven scenarios reflect levels of intensity ranging from once in a lifetime screening at age 35 (Scenario 1) to HPV-based screening at 5-year intervals in all but the Low Income (LI) countries, where VIA would be offered every three years for women of screening age (Scenario 7) (Table 1). For countries in the LMI2 income tier and higher, strategies may vary by the presence of an existing Pap program if coverage exceeds 40% [30,31]. Scenarios 4 and 6 are identical to Scenarios 3 and 5 (respectively), with the exception that Pap testing has been replaced by HPV-based testing in countries with Pap programs. With the exception of countries with existing Pap programs with coverage greater than 40%, screening strategies did not depend on existing screening modality or coverage level in a given country, but rather on income tier.

**Table 1:** Screening strategies, by income tier.^a^

Income tier	Existing Cytology-based Program^b^	Screening scenario						
		Scenario 1: Onetime screening	Scenario 2: Minimal intensity	Scenario 3: Lower intensity w/Pap	Scenario 4: Lower intensity w/o Pap	Scenario 5: Higher intensity w/Pap	Scenario 6: Higher intensity w/o Pap	Scenario 7: HPV-based screening
Low income (LI) (< $1045)	No	VIA 1x	VIA Q10	VIA Q5	VIA Q5	VIA Q3	VIA Q3	VIA Q3
Lower-middle income 1 (LMI1) ($1046–$2585)	No	VIA 1x	VIA Q10	VIA Q5	VIA Q5	VIA Q3	VIA Q3	HPV Q5
Lower-middle income 2 (LMI2) ($2586–$4125)	No	VIA 1x	VIA Q10	VIA Q3	VIA Q3	HPV Q5	HPV Q5	HPV Q5
	Yes	PAP 1x	PAP Q10	PAP Q5	HPV Q5	PAP Q3	HPV Q5	HPV Q5
Upper-middle income 1 (UMI1) ($4126–$8435)	No	HPV 1x	HPV Q10	HPV Q5	HPV Q5	HPV-VIA Q5	HPV-VIA Q5	HPV-VIA Q5
	Yes	PAP 1x	PAP Q10	PAP Q5	HPV Q5	PAP Q3	HPV-VIA Q5	HPV-VIA Q5
Upper-middle income 2 (UMI2) ($8436–$12745)	No	HPV-VIA 1x	HPV-VIA Q10	HPV-VIA Q5	HPV-VIA Q5	HPV-VIA Q5	HPV-VIA Q5	HPV-VIA Q5
	Yes	PAP 1x	PAP Q10	PAP Q5	HPV-VIA Q5	PAP Q3	HPV-VIA Q5	HPV-VIA Q5

^a^ HPV: human papillomavirus testing; HPV-VIA: HPV testing with visual inspection triage; PAP: Pap testing; VIA: visual inspection with acetic acid; 1x: Once in a lifetime at age 35 years; Q10: screening at 10-year interval (at age 30, 40 years); Q5: screening at 5-year interval (at age 30, 35, 40, 45 years); Q3: screening at 3-year interval (at age 30, 33, 36, 39, 42, 45, 48 years); $: 2013 US$.

^b^ Existing cytology programs with >40% coverage (includes Argentina, Brazil, Colombia, Costa Rica, Dominican Republic, El Salvador, Hungary, Kazakhstan, Mexico, Paraguay, Peru, Ukraine) [30,31].

We assumed the following three roll-out strategies for both vaccination and screening: 1) 100% immediate coverage from 2015 to 2024 (Immediate Roll-out); 2) 20% coverage in 2015, 40% coverage in 2016, 60% coverage in 2017, 80% coverage in 2018, and 100% coverage of the target population from 2019 to 2024 (5-year Roll-out); and 3) 10% coverage in 2015, 20% coverage in 2016, 30% coverage in 2017, 40% coverage in 2018, 50% coverage in 2019, 60% coverage in 2020, 70% coverage in 2021, 80% coverage in 2022, 90% coverage in 2023, and 100% coverage of the target population in 2024 (10-year Roll-out). We assumed these scenarios irrespective of a country’s existing immunization and screening program coverage.

To provide an upper bound on cost estimates, we assumed no loss-to-follow-up of screen-positive women between visits for confirmatory diagnostic testing and/or treatment. Because management algorithms for screen-positive women vary by setting, we made the following simplifying assumptions across all countries: 1) visual inspection with acetic acid (VIA) and HPV testing are followed by cryotherapy for eligible screen-positive women (i.e., a screen-and-treat approach); 2) HPV testing with VIA triage for HPV-positive women (HPV-VIA) is followed by cryotherapy for eligible women who screen positive on VIA (i.e., a screen-and-treat approach); 3) a proportion of women who screen positive with HPV and/or VIA testing are deemed ineligible for immediate treatment (5% of women with no lesion, 15% of women with CIN1, and 25% of women with CIN2/3) and require a colposcopy/biopsy; 5) Pap testing is followed by colposcopy/biopsy for all screen-positive women; 6) women with histologically confirmed CIN1 receive cryotherapy, while women with histologically confirmed CIN2/3 receive LEEP.

Screening test performance parameters are displayed in **Table E in** S1 File. To capture costs associated with screening, diagnosis, and treatment of precancer, CERVIVAC estimates the number of true positives (women with CIN1 or CIN2/3 who screen positive) and the number of false positives (women with no lesion who screen positive) based on screening test performance and the prevalence of oncogenic HPV, CIN1 and CIN23. Due to limited data and variation in colposcopy performance by setting, we have assumed perfect colposcopy (i.e., 100% sensitivity and specificity at the CIN1+ threshold) in each country.

To account for reduced lesion prevalence in scenarios with repeated screening, we derived “attenuation factors” for both CIN1 and CIN2/3 lesions. We simulated VIA, Pap, and HPV testing in the calibrated microsimulation models at 100% coverage of the target group of women (aged 30 to 49 years), for each screening frequency considered. From model output we calculated the percent reduction in CIN1 and CIN2/3 lesions immediately prior to each screening after the first. This percent reduction was averaged across the number of subsequent screenings, and this average was subtracted from one to generate the “attenuation factor” associated with a given screening modality and frequency. Thus, the attenuation factor is the ratio between the prevalence of lesions at the first screening visit and the prevalence of lesions expected at all subsequent screening visits. Attenuation factors are displayed in **Table F in** S1 File.

### Cost input data

For vaccination, we included HPV vaccination delivery costs and the price of the vaccine under three scenarios in which we varied the per-dose price of the vaccine by income tier. Sources of HPV vaccine price information included Gavi, the Vaccine Alliance, PAHO Revolving Fund (RF) and a report from Médecins Sans Frontières (Table 2). We assumed Gavi-eligible countries would have access to the Gavi-negotiated prices in all years under all scenarios. Similarly, we assumed all Latin America and Caribbean (LAC) region countries and South Africa would access the PAHO RF price in all years under all scenarios, because these countries have access to the PAHO RF (though not all use it). Prices for non-Gavi, non-LAC countries were based on country income tiers. Under each scenario we assumed that vaccine pricing would remain constant between 2015 and 2024, and that countries would remain in the same income tier. The most optimistic (lowest cost) Scenario A is a two-tier scheme where all Gavi countries access the Gavi price of US$4.55 per dose, all non-Gavi LMI1 countries access the Gavi price as well, and all non-Gavi countries in income tier LMI2 or higher access a price equal to the RF price of US$13.79 per dose. In alternative scenarios, prices for non-Gavi non-RF countries were assumed to be higher (Scenarios B and C).

**Table 2:** HPV vaccine price per dose scenarios, by income tier.^a^

Income tier	Vaccination scenario^b^		
	Scenario A	Scenario B	Scenario C
Low income (LI) (< $1045)	4.55	4.55	4.55
Lower-middle income 1 (LMI1) ($1046–$2585)	4.55	13.79	13.79
Lower-middle income 2 (LMI2) ($2586–$4125)	13.79	27.58	27.58
Upper-middle income 1 (UMI1) ($4126–$8435)	13.79	40	40
Upper-middle income 2 (UMI2) ($8436–$12745)	13.79	40	70

^a^ $: 2013 US$. We assumed Gavi-eligible countries would have access to the Gavi-negotiated prices in all years under all scenarios. Similarly, we assumed all Latin America and Caribbean (LAC) region countries and South Africa would access the PAHO Revolving Fund price in all years under all scenarios, because these countries have access to the PAHO Revolving Fund (though not all use it). Prices for non-Gavi, non-LAC countries were based on country income tiers.

^b^ Vaccination Scenario A is a two-tier scheme where all Gavi-eligible and non-Gavi-eligible LMI1 countries access the Gavi price of US$4.55 per dose, while all non-Gavi countries in income tier LMI2 or higher access a price equal to the PAHO Revolving Fund price of US$13.79 per dose. Vaccination Scenario B applies the Gavi price to LI countries only, and the PAHO Revolving Fund price to LMI1 countries; LMI2 countries are assumed to receive twice the Revolving Fund price, while UMI countries receive the best UMI price of US$40 per dose according to Médecins Sans Frontières. Vaccination Scenario C is similar to scenario B except UMI2 countries are subject to a higher price of US$70 per dose.

We identified data on HPV vaccination delivery cost per dose from the published literature and restricted estimates to economic costs, defined as the cost of all resources used regardless of payer [32–34]; unlike financial costs, economic costs include the salaries of health personnel which have already been paid for prior to initiation of an HPV vaccination program, but still represent an opportunity cost. When possible, we excluded start-up costs which would only be relevant within the initial years of a program. We considered all delivery mechanisms, including school-based, health center-based, and integrated outreach. All costs were converted to 2013 US$ using GDP deflators and exchange rates (**Table G in** S1 File). We assumed that delivery costs did not vary with vaccination coverage.

For screening-related costs, we included direct medical costs associated with screening, diagnosis, and treatment of precancerous lesions. Procedures and assumed location of service delivery are presented in **Table H in** S1 File. To estimate the unit cost of each procedure, we identified available data from the published literature [9,35], unpublished data from PATH's START-UP demonstration projects [3,36], and data from a pilot study in El Salvador [37]; as such, primary data were available for the following countries: Ghana, El Salvador, India (n = 3 studies), Kenya, Nicaragua, Peru, Uganda, South Africa, and Thailand. All unit costs were converted to 2013 US$ using GDP deflators and exchange rates (**Table I in** S1 File)[11].

To extrapolate primary data estimates for each vaccination and screening procedure to all countries, we obtained the unit cost of a primary health center visit and a secondary outpatient hospital visit in each country from the WHO-CHOICE costing tool [38]; the latest available local currency unit estimates from 2008 were converted into 2013 US$ using GDP deflators and exchange rates. We took the ratio of the WHO-CHOICE facility cost in Country X to the WHO-CHOICE facility cost in a country with primary data for a given procedure. We multiplied this ratio by the cost of the procedure in the primary data to obtain an estimate of the procedure cost in Country X. We repeated this calculation for each primary data estimate associated with a given procedure, and then took the average of extrapolated values to use in analysis. By incorporating WHO-CHOICE data, these extrapolated values explicitly take into account the high correlation between a country's GDP per capita and health care costs. We assumed that the HPV test was a tradable good with a standardized value of US$5 across all settings, and thus did not apply WHO-CHOICE facility ratios to this value to obtain HPV screening costs; rather, we applied WHO-CHOICE ratios to the other direct medical cost components of HPV testing (e.g., staff time, laboratory processing, other supplies) and added the US$5 test cost afterward. We performed a sensitivity analysis in which we assumed the cost of the HPV test ranged from US$2.50 to US$7.50, to estimate the potential impact of bulk purchasing of HPV tests or a potentially higher market price. Results for the average HPV vaccine delivery cost per dose, by income tier and Gavi eligibility, are shown in **Table J in** S1 File; unit costs by screening, diagnostic, and precancer treatment procedure for each income tier are shown in **Table K in** S1 File. Unit costs for each procedure are plotted against 2013 GDP per capita in **Figure A in** S1 File.

### Cost per woman of screening age

To calculate the average cost per woman of screening age under each screening scenario, we assumed the total population size remained static over 10 years and was represented by the total population of women aged 30 to 49 years in 2015. We divided the total cost of screening, diagnostic confirmation, and treatment of precancer at full coverage from 2015 to 2024 by this total population size of 30 to 49 year old women in 2015 to estimate the cost per woman of screening age during the period of interest.

## Results

The total cost of HPV vaccination from 2015 to 2024 by income tier and World Bank region, for each roll-out strategy and vaccine pricing scenario, is presented in Table 3 (undiscounted; discounted results are presented in **Table L in** S1 File). The total cost for immediate roll-out in the optimistic pricing scenario (Scenario A) is US$15.5 billion, or about US$1.55 billion per year. About 58% of this cost is for the vaccine product itself. This scenario would vaccinate 513 million girls over 10 years at an average cost of US$30.19 per girl, ranging from US$12 per girl in LI settings to US$59 per girl in UMI2 settings. With less favorable vaccine pricing (Scenario C), the cost of immediate roll-out could be as much as US$24.2 billion, and the vaccine product itself would represent 73% of this cost. A 5-year roll-out would reach about 415 million girls and cost US$12.5 billion in the most optimistic pricing scenario (Scenario A). A 10-year roll-out would reach 286 million girls and cost US$8.6 billion in the most optimistic pricing scenario (Scenario A).

**Table 3:** Total undiscounted cost of HPV vaccination from 2015 to 2024, by income tier, World Bank region, and vaccination scenario (2013 US$, billions).^a^

Income tier or World Bank region	Number of 10-year old girls in 2015, LMIC (% of total)	Vaccination scenario		
		A	B	C
***Immediate roll-out***^b^, ***cost (% of total costs)***				
**TOTAL**	49,743,665	**$15.5 B**	**$23.5 B**	**$24.2 B**
***By Income Tier***				
**LI**	9,868,831 (20%)	$1.3 B (8)	$1.3 B (5)	$1.3 B (5)
**LMI1**	16,988,122 (34%)	$2.6 B (17)	$2.8 B (12)	$2.8 B (11)
**LMI2**	7,610,480 (15%)	$3.4 B (22)	$5.5 B (23)	$5.5 B (23)
**UMI1**	10,427,821 (21%)	$5.6 B (36)	$10.6 B (45)	$10.6 B (44)
**UMI2**	4,848,411 (10%)	$2.7 B (17)	$3.3 B (14)	$4.1 B (17)
***By World Region***				
**EAP**	12,545,391 (25%)	$5.8 B (37)	$11.2 B (48)	$11.4 B (47)
**ECA**	1,848,914 (4%)	$1.0 B (6)	$1.8 B (7)	$2.3 B (10)
**LAC**	5,162,675 (10%)	$2.7 B (17)	$2.8 B (12)	$2.8 B (11)
**MENA**	1,909,780 (4%)	$0.8 B (5)	$1.5 B (6)	$1.5 B (6)
**SA**	15,897,310 (32%)	$2.4 B (15)	$2.4 B (10)	$2.4 B (10)
**SSA**	12,379,595 (25%)	$2.9 B (19)	$3.9 B (17)	$3.9 B (16)
***By Gavi eligibility***				
**Gavi-eligible**^c^	26,216,170 (53%)	$3.8 B (24)	$3.8 B (16)	$3.8 B (16)
**Non-Gavi eligible**	23,527,495 (47%)	$11.7 B (76)	$19.7 B (84)	$20.4 B (84)
***5-year roll-out***^b^, ***cost (% of total costs)***				
**TOTAL**	49,743,665	**$12.5 B**	**$19 B**	**$19.5 B**
***By Income Tier***				
**LI**	9,868,831 (20%)	$1 B (8)	$1 B (5)	$1 B (5)
**LMI1**	16,988,122 (34%)	$2.1 B (17)	$2.2 B (12)	$2.2 B (11)
**LMI2**	7,610,480 (15%)	$2.7 B (22)	$4.5 B (24)	$4.5 B (23)
**UMI1**	10,427,821 (21%)	$4.5 B (36)	$8.6 B (45)	$8.6 B (44)
**UMI2**	4,848,411 (10%)	$2.1 B (17)	$2.6 B (14)	$3.2 B (17)
***By World Region***				
**EAP**	12,545,391 (25%)	$4.6 B (37)	$9.1 B (48)	$9.2 B (47)
**ECA**	1,848,914 (4%)	$0.8 B (6)	$1.4 B (7)	$1.9 B (10)
**LAC**	5,162,675 (10%)	$2.1 B (17)	$2.2 B (12)	$2.2 B (11)
**MENA**	1,909,780 (4%)	$0.7 B (5)	$1.2 B (6)	$1.2 B (6)
**SA**	15,897,310 (32%)	$1.9 B (15)	$1.9 B (10)	$1.9 B (10)
**SSA**	12,379,595 (25%)	$2.4 B (19)	$3.2 B (17)	$3.2 B (16)
***By Gavi eligibility***				
**Gavi-eligible**^c^	26,216,170 (53%)	$3 B (24)	$3 B (16)	$3 B (16)
**Non-Gavi eligible**	23,527,495 (47%)	$9.5 B (76)	$15.9 B (84)	$16.5 B (84)
***10-year roll-out***^b^, ***cost (% of total costs)***				
**TOTAL**	49,743,665	**$8.6 B**	**$13.1 B**	**$13.5 B**
***By Income Tier***				
**LI**	9,868,831 (20%)	$0.7 B (8)	$0.7 B (5)	$0.7 B (5)
**LMI1**	16,988,122 (34%)	$1.5 B (17)	$1.5 B (12)	$1.5 B (11)
**LMI2**	7,610,480 (15%)	$1.9 B (22)	$3.1 B (24)	$3.1 B (23)
**UMI1**	10,427,821 (21%)	$3.1 B (36)	$6.0 B (45)	$6.0 B (44)
**UMI2**	4,848,411 (10%)	$1.5 B (17)	$1.8 B (14)	$2.2 B (16)
***By World Region***				
**EAP**	12,545,391 (25%)	$3.2 B (37)	$6.3 B (48)	$6.4 B (47)
**ECA**	1,848,914 (4%)	$0.5 B (6)	$1 B (7)	$1.3 B (9)
**LAC**	5,162,675 (10%)	$1.5 B (17)	$1.5 B (11)	$1.5 B (11)
**MENA**	1,909,780 (4%)	$0.5 B (5)	$0.8 B (6)	$0.8 B (6)
**SA**	15,897,310 (32%)	$1.3 B (15)	$1.3 B (10)	$1.3 B (10)
**SSA**	12,379,595 (25%)	$1.7 B (19)	$2.2 B (17)	$2.2 B (16)
***By Gavi eligibility***				
**Gavi-eligible**^c^	26,216,170 (53%)	$2.1 B (24)	$2.1 B (16)	$2.1 B (16)
**Non-Gavi eligible**	23,527,495 (47%)	$6.5 B (76)	$11 B (84)	$11.4 B (84)

^a^ Gavi: Gavi, the Vaccine Alliance; LMIC: low- and middle-income countries; LI: Low income; LMI1: Lower-middle income tier 1; LMI2: Lower-middle income tier 2; UMI1: Upper-middle income tier 1; UMI2: Upper-middle income tier 2; EAP: East Asia & Pacific; ECA: Europe & Central Asia; LAC: Latin America & Caribbean; MENA: Middle East & North Africa; SA: South Asia; SSA: Sub-Saharan Africa.

^b^ Immediate roll-out: Full coverage (100%) of the target population from 2015 to 2024; Rapid roll-out: 20% coverage in 2015, 40% coverage in 2016, 60% coverage in 2017, 80% coverage in 2018, and 100% coverage of the target population from 2019 to 2024; Gradual roll-out: 10% coverage in 2015, 20% coverage in 2016, 30% coverage in 2017, 40% coverage in 2018, 50% coverage in 2019, 60% coverage in 2020, 70% coverage in 2021, 80% coverage in 2022, 90% coverage in 2023, and 100% coverage of the target population in 2024.

^**c**^ Eligible for assistance from Gavi, the Vaccine Alliance, in 2014. See **Table A in the** S1 File for listing of 43 countries.

The total cost of cervical cancer screening, diagnostic testing, and treatment of precancerous lesions from 2015 to 2024 by income tier and World Bank region, for each roll-out strategy and screening scenario, is presented in Table 4 (undiscounted; discounted results are presented in **Table M in** S1 File). With immediate roll-out, Scenario 1 (screening once at age 35) was the least costly (US$9.0 billion). Scenario 2 (screening at ages 30 and 40) was the next least costly, at a total cost of US$17.6 billion. Scenario 5 (high intensity screening with Pap) was the most costly due to frequent Pap screening every three years in countries with existing cytology programs (US$42.3 billion); Scenario 6 (high intensity screening without Pap), in which triennial Pap screening was replaced with HPV or HPV-VIA testing every 5 years, yielded a lower total cost (US$29.4 billion). The vast majority of savings (78%) associated with switching from Scenario 5 to Scenario 6 were in UMI2 countries, which include several populous countries (i.e., Brazil and Mexico) that were assumed to use HPV-VIA testing in Scenario 6. Despite the additional costs of a triage test with HPV-VIA, this screening test system yielded substantial savings in diagnostic and treatment costs relative to Pap screening.

**Table 4:** Total undiscounted cost of cervical cancer screening and treatment from 2015 to 2024, by income tier, World Bank region, and screening scenario (2013 US$, millions).^a^

Income tier or World Bank region	Number of women aged 30–49 years in 2015, LMIC (% of total)	Screening scenario						
		1	2	3	4	5	6	7
***Immediate roll-out***^b^, ***cost (% of total costs)***								
**TOTAL**	760,402,598	**$9.0 B**	**$17.6 B**	**$36.6 B**	**$31.1 B**	**$42.3 B**	**$29.4 B**	**$29.2 B**
***By Income Tier***								
**LI**	84,739,921 (11%)	$0.2 B (3)	$0.5 B (3)	$0.8 B 2)	$0.8 B (3)	$1.4 B (3)	$1.4 B (5)	$1.4 B (5)
**LMI1**	230,936,378 (30%)	$1.0 B (11)	$2.0 B (12)	$3.8 B (10)	$3.8 B (12)	$6.4 B (15)	$6.4 B (22)	$6.2 B (21)
**LMI2**	94,895,600 (12%)	$1.1 B (12)	$2.0 B (12)	$6.1 B (17)	$6.0 B (20)	$5.7 B (13)	$5.1 B (18)	$5.1 B (18)
**UMI1**	263,137,970 (35%)	$4.1 B (45)	$8.0 B (46)	$16.0 B (46)	$15.3 B (49)	$13.5 B (32)	$11.2 B (38)	$11.2 B (39)
**UMI2**	86,992,729 (11%)	$2.5 B (28)	$5.1 B (29)	$9.8 B (26)	$5.2 B (16)	$15.3 B (36)	$5.2 B (18)	$5.2 B (18)
***By World Region***								
**EAP**	295,355,455 (39%)	$3.8 B (42)	$6.9 B (39)	$15.4 B (42)	$15.4 B (50)	$12.2 B (29)	$12.2 B (41)	$12.1 B (41)
**ECA**	39,367,836 (5%)	$0.8 B (9)	$1.6 B (9)	$3.1 B (8)	$2.6 B (8)	$3.8 B (9)	$2.5 B (8)	$2.5 B (9)
**LAC**	82,566,777 (11%)	$2.6 B (29)	$5.2 B (30)	$10.0 B (27)	$5.0 B (16)	$16.3 B (39)	$4.8 B (16)	$4.8 B (16)
**MENA**	27,105,360 (4%)	$0.3 B (3)	$0.6 B (4)	$1.6 B (4)	$1.6 B (5)	$1.2 B (3)	$1.2 B (4)	$1.2 B (4)
**SA**	220,923,612 (29%)	$0.9 B (10)	$1.8 B (10)	$3.5 B (9)	$3.5 B (11)	$5.6 B (13)	$5.6 B (19)	$5.4 B (19)
**SSA**	95,083,558 (13%)	$0.7 B (8)	$1.5 B (8)	$3.1 B (8)	$3.1 B (10)	$3.2 B (8)	$3.2 B (11)	$3.3 B (11)
***5-year roll-out***^b^, ***cost (% of total costs)***								
**TOTAL**	760,402,598	**$7.4 B**	**$14.1 B**	**$29.4 B**	**$25.0 B**	**$34.0 B**	**$23.7 B**	**$23.5 B**
***By Income Tier***								
**LI**	84,739,921 (11%)	$0.2 B (3)	$0.4 B (3)	$0.7 B (2)	$0.7 B (3)	$1.1 B (3)	$1.1 B (5)	$1.1 B (5)
**LMI1**	230,936,378 (30%)	$0.8 B (11)	$1.6 B (12)	$3.1B (11)	$3.1 B (12)	$5.2 B (15)	$5.2 B (22)	$5.1 B (22)
**LMI2**	94,895,600 (12%)	$0.9 B (12)	$1.6 B (12)	$4.9 B (17)	$4.8 B (20)	$4.6 B (13)	$4.2 B (18)	$4.2 B (18)
**UMI1**	263,137,970 (35%)	$3.4 B (46)	$6.4 B (45)	$12.8 B (43)	$12.2 B (49)	$10.8 B (31)	$9.0 B (38)	$9.0 B (38)
**UMI2**	86,992,729 (11%)	$2.0 B (27)	$4.1 B (29)	$7.9 B (26)	$4.2 B (16)	$12.2 B (36)	$4.2 B (18)	$4.2 B (18)
***By World Region***								
**EAP**	295,355,455 (39%)	$3.1 B (42)	$5.5 B (39)	$12.3 B (42)	$12.3 B (49)	$9.7 B (29)	$9.7 B (41)	$9.6 B (41)
**ECA**	39,367,836 (5%)	$0.6 B (9)	$1.3 B (9)	$2.4 B (8)	$2.1 B (8)	$3.1 B (9)	$2.0 B (8)	$2.0 B (9)
**LAC**	82,566,777 (11%)	$2.1 B (28)	$4.2 B (30)	$8.0 B (27)	$4.0 B (16)	$13.1 B (38)	$3.8 B (16)	$3.8 B (16)
**MENA**	27,105,360 (4%)	$0.2 B (3)	$0.5 B (4)	$1.3 B (4)	$1.3 B (5)	$1.0 B (3)	$1.0 B (4)	$1.0 B (4)
**SA**	220,923,612 (29%)	$0.7 B (10)	$1.4 B (10)	$2.8 B (10)	$2.8 B (11)	$4.5 B (13)	$4.5 B (19)	$4.4 B (19)
**SSA**	95,083,558 (13%)	$0.6 B (8)	$1.2 B (9)	$2.5 B (9)	$2.5 B (10)	$2.6 B (8)	$2.6 B (11)	$2.7 B (11)
***10-year roll-out***^b^, ***cost (% of total costs)***								
**TOTAL**	760,402,598	**$5.1 B**	**$9.7 B**	**$20.3 B**	**$17.2 B**	**$23.4 B**	**$16.3 B**	**$16.2 B**
***By Income Tier***								
**LI**	84,739,921 (11%)	$0.1 B (3)	$0.3 B (3)	$0.5 B (2)	$0.5 B (3)	$0.8 B (3)	$0.8 B (5)	$0.8 B (5)
**LMI1**	230,936,378 (30%)	$0.6 B (11)	$1.1 B (12)	$2.2 B (11)	$2.2 B (13)	$3.6 B (15)	$3.6 B (22)	$3.5 B (22)
**LMI2**	94,895,600 (12%)	$0.6 B (12)	$1.1 B (12)	$3.4 B (17)	$3.4 B (20)	$3.2 B (13)	$2.9 B (18)	$2.9 B (18)
**UMI1**	263,137,970 (35%)	$2.4 B (47)	$4.3 B (45)	$8.8 B (43)	$8.4 B (49)	$7.4 B (31)	$6.2 B (38)	$6.2 B (38)
**UMI2**	86,992,729 (11%)	$1.4 B (27)	$2.8 B (29)	$5.4 B (26)	$2.9 B (16)	$8.4 B (36)	$2.9 B (18)	$2.9 B (18)
***By World Region***								
**EAP**	295,355,455 (39%)	$2.2 B (43)	$3.8 B (39)	$8.5 B (42)	$8.5 B (49)	$6.7 B (29)	$6.7 B (41)	$6.6 B (41)
**ECA**	39,367,836 (5%)	$0.4 B (9)	$0.9 B (9)	$1.7 B (8)	$1.4 B (8)	$2.1 B (9)	$1.4 B (8)	$1.4 B (9)
**LAC**	82,566,777 (11%)	$1.4 B (28)	$2.9 B (30)	$5.5 B (27)	$2.8 B (16)	$9.0 B (38)	$2.6 B (16)	$2.6 B (16)
**MENA**	27,105,360 (4%)	$0.2 B (3)	$0.4 B (4)	$0.9 B (4)	$0.9 B (5)	$0.7 B (3)	$0.7 B (4)	$0.7 B (4)
**SA**	220,923,612 (29%)	$0.5 B (10)	$1.0 B (10)	$2.0 B (10)	$2.0 B (11)	$3.1 B (13)	$3.1 B (19)	$3.0 B (19)
**SSA**	95,083,558 (13%)	$0.4 B (8)	$0.8 B (9)	$1.8 B (9)	$1.8 B (10)	$1.8 B (8)	$1.8 B (11)	$1.9 B (11)

^a^ LMIC: low- and middle-income countries; LI: Low income; LMI1: Lower-middle income tier 1; LMI2: Lower-middle income tier 2; UMI1: Upper-middle income tier 1; UMI2: Upper-middle income tier 2; EAP: East Asia & Pacific; ECA: Europe & Central Asia; LAC: Latin America & Caribbean; MENA: Middle East & North Africa; SA: South Asia; SSA: Sub-Saharan Africa.

^b^ Immediate roll-out: Full coverage (100%) of the target population from 2015 to 2024; 5-year roll-out: 20% coverage in 2015, 40% coverage in 2016, 60% coverage in 2017, 80% coverage in 2018, and 100% coverage of the target population from 2019 to 2024; 10-year roll-out: 10% coverage in 2015, 20% coverage in 2016, 30% coverage in 2017, 40% coverage in 2018, 50% coverage in 2019, 60% coverage in 2020, 70% coverage in 2021, 80% coverage in 2022, 90% coverage in 2023, and 100% coverage of the target population in 2024.

**Fig 1:**
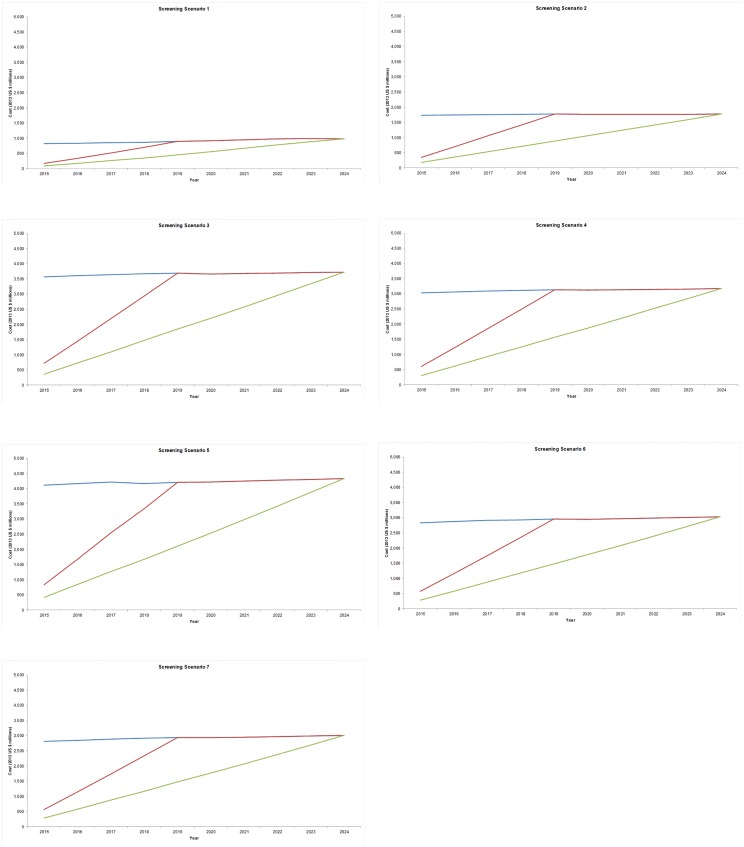
Screening and treatment costs by screening scenario, year and roll-out strategy (2013 US$, millions). Each panel displays the cost (y-axis, 2013 US$) in each year (x-axis) associated with immediate (blue line), 5-year (red line), and 10-year (green line) roll-out in each screening and treatment scenario: Scenario 1, screening once at age 35 (panel A); Scenario 2, screening at a 10-year interval at age 30 and 40(panel B); Scenario 3, low intensity screening, with Pap testing in countries with existing cytology programs (panel C); Scenario 4, low intensity screening without Pap testing (panel D); Scenario 5, high intensity screening with Pap testing in countries with existing cytology programs (panel E); Scenario 6, high intensity screening without Pap testing (panel F); and Scenario 7, HPV-based screening in all but the lowest income tier (panel G). Roll-out assumptions are described in the Methods.

For all scenarios, immediate roll-out yielded the highest total costs over the 10-year period, while 5-year roll-out reduced total costs by approximately 20% as scaling of screening and treatment programs was spread over the first 5 years, and gradual roll-out over 10 years reduced costs by approximately 45%. While the cost per year changes little once full coverage of the target population is achieved, the yearly cost varies substantially by screening scenario (Fig 1). Under Scenario 1 (screening once at age 35), yearly costs of 10-year roll-out range from US$82 million in 2015 to US$975 million in 2024, totaling US$5.1 billion for the 10-year period during which more than 252 million women would be screened (relative to US$9.0 billion for immediate roll-out, during which more than 440 million women would be screened).

The greatest proportion of the LMIC screening population resides in the UMI1 income tier (35%), which account for 32% (Scenario 5) to 46% (Scenarios 2 and 3) of total costs. While 30% of the LMIC screening population resides in the LMI1 income tier, these countries represent only 12% of total costs under Scenario 1 (screening once at age 35) with immediate roll-out, due to the use of the least costly screening test (i.e., VIA) and the relationship between direct medical costs and GDP per capita. With a shift to HPV testing in LMI1 under Scenario 7 (HPV-based screening at 5-year intervals), this income tier still represents only 21% of total costs with full immediate coverage. The LI countries, comprising 11% of the LMIC population, incur only 2% to 5% of the total screening and treatment costs under any scenario, due to the exclusive use of VIA in these settings and low direct medical costs relative to other income tiers.

When stratified by World Bank region, the largest component of the LMIC population resides in the East Asia and Pacific Region (39%), followed by South Asia (29%). Assuming immediate roll-out, screening- and treatment-related costs in the East Asia and Pacific Region account for 29% (Scenario 5) to 50% (Scenario 4) of total costs; South Asia accounts for only 9% (Scenario 3) to 19% (Scenario 6) of total screening and treatment-related costs, due to the primarily LI and LMI1 countries that comprise this region. Screening- and treatment-related costs in Sub-Saharan Africa (which is home to 13% of women of screening age in LMIC) account for 8% (Scenarios 1, 2, 3, and 5) to 11% (Scenarios 6 and 7) of total costs.

**Fig 2:**
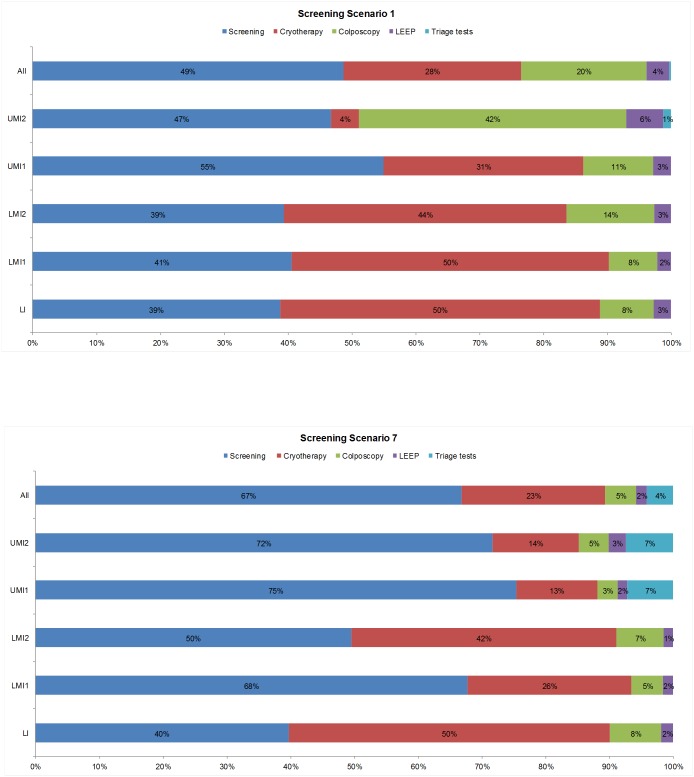
Distribution of cervical cancer screening, diagnostic, and treatment costs for Screening Scenario 1 (once in a lifetime screening) and Screening Scenario 7 (HPV-based screening in Lower-middle income tier 1 and above), immediate roll-out. Bars display the proportion of total costs associated with each procedure for Scenario 1, screening once at age 35 (upper panel) and Scenario 7, HPV-based screening in all but the lowest income tier (lower panel): screening tests (blue); cryotherapy (red); colposcopy/biopsy (green); loop electrosurgical excision procedure (LEEP) (dark purple), and triage tests (turquoise). The distribution of costs is presented for all countries (top bar) and by income tier.

The relative distribution of screening, diagnostic, and treatment costs is displayed in Fig 2. Scenarios 1 (screening once at age 35) and 7 (HPV-based screening at 5-year intervals in all but LI countries) represent the extremes of this distribution. In Scenario 1, 49% of total costs are attributable to screening tests. Because HPV-VIA is offered only in UMI2 countries without existing Pap programs, triage tests account for <1% of total costs. The reliance on Pap screening in the 12 countries with existing programs results in 20% of total costs being spent on colposcopy. Cryotherapy accounts for 28% of costs, and LEEP for 4%. The shift to HPV-based testing in all but LI countries under Scenario 7 raises the relative contribution of screening costs to 67%, with the relative share of LMI1 and LMI2 countries increasing as VIA (and Pap, in El Salvador, Paraguay, and Ukraine) is replaced by HPV testing in these income tiers. The proportional cost of triage tests increases slightly to 4%, as HPV-VIA is adopted in all UMI countries, while the reduced reliance on Pap testing leads to a reduced contribution of colposcopy to total costs (5%). Cryotherapy and LEEP account for 25% of total costs, as the number of women treated decreases as a result of HPV-VIA triage testing.

The average cost per woman of screening age for the 10-year period from 2015 to 2024, assuming full coverage of the target population (i.e., immediate roll-out), is displayed in Table 5. Under Scenario 1 (screening once at age 35), the cost per woman of screening age is US$11.87; this increases to US$23.13 when women are screened at ages 30 and 40 years (Scenario 2). The highest cost per woman of screening age is associated with Scenario 5 (higher intensity screening with Pap), at US$55.59. With a shift toward HPV-based screening, this cost is reduced to US$38.65 when VIA is still offered in LI and LMI1 countries (Scenario 6). A shift to HPV-based screening in all but the LI countries (Scenario 7) has only a marginal impact on the cost per woman of screening age, which falls slightly to US$38.41. When we consider time preferences and apply a discount rate of 3% per year, the cost per woman of screening age is roughly 15% lower under each screening scenario.

**Table 5:** Cost, over 10 years, per woman of screening age in 2015, by screening scenario (2013 US$).^a^

	Screening scenario						
Income tier	1	2	3	4	5	6	7
**Cost per woman of screening age, undiscounted**							
**All**	11.87	23.13	48.15	40.93	55.59	38.65	38.41
**LI**	2.78	5.48	10.02	10.02	16.44	16.44	16.44
**LMI1**	4.49	8.79	16.64	16.64	27.87	27.87	27.06
**LMI2**	11.21	21.31	64.28	63.19	59.71	54.19	54.19
**UMI1**	15.74	30.32	60.90	58.00	51.41	42.74	42.74
**UMI2**	29.30	58.54	112.68	59.51	175.33	59.51	59.51
**Cost per woman of screening age, discounted**							
**All**	10.07	19.72	41.03	34.87	47.36	32.92	32.71
**LI**	2.35	4.64	8.49	8.49	13.93	13.93	13.93
**LMI1**	3.81	7.47	14.13	14.13	23.68	23.68	22.98
**LMI2**	9.54	18.13	54.69	53.76	50.82	46.10	46.10
**UMI1**	13.31	25.90	51.97	49.50	43.87	36.48	36.48
**UMI2**	24.96	49.90	96.05	50.71	149.49	50.71	50.71

^a^: LI: Low income; LMI1: Lower-middle income tier 1; LMI2: Lower-middle income tier 2; UMI1: Upper-middle income tier 1; UMI2: Upper-middle income tier 2. Cost per woman of screening age is defined as the total cost of screening, diagnostic confirmation, and treatment of precancer at full coverage from 2015 to 2024 divided by the total population of 30 to 49 year old women in 2015. Calculations assume immediate roll-out.

Providing both vaccination and screening in a comprehensive cervical cancer prevention program would cost US$13.7 billion with a 10-year roll-out strategy, favorable vaccine pricing (Scenario A), and screening once at age 35 (Scenario 1); this would cover the cost of vaccinating 286 million girls and screening more than 250 million women. Under the least favorable vaccine pricing (Scenario C), this total cost would rise to US$18.6 billion. Immediate roll-out of vaccination and Scenario 7 (HPV-based screening at 5-year intervals in LMI1 and above, and VIA at 3-year intervals in LI settings) would cost US$44.7 billion over the 10 years in the most favorable vaccine pricing scenario (A), and US$53.4 billion in the least favorable vaccine pricing scenario (C).

We performed a sensitivity analysis in which the assumed cost of the HPV test was varied from US$2.50 to US$7.50 in Scenario 7 (HPV-based screening in all but LI countries), relative to the base case value of US$5. At an HPV test cost of US$2.50, total costs of screening associated with immediate roll-out were reduced from US$29.2 billion to US$25.5 billion. The cost per woman of screening age fell from US$38.41 to US$33.58. At a higher HPV test cost of US$7.50, the total cost of screening with immediate roll-out increased to US$32.9 billion, and the cost per woman of screening age rose to US$43.23.

## Discussion

To the best of our knowledge, this study provides the first estimate of a global cost for cervical cancer prevention in LMIC, including HPV vaccination of young adolescent girls and screening and preventive treatment of women aged 30 to 49 years. We considered multiple policy scenarios—3 alternate vaccine pricing assumptions, 7 screening test and frequency assumptions, and 3 roll-out strategies—to capture a reasonable range of costs associated with primary and secondary prevention of cervical cancer in LMIC. While the immediate roll-out strategy is not realistic, we present it as an upper bound that might represent spending for a fully scaled program. We found that from years 2015 to 2024 HPV vaccination would cost from US$8.6 billion to US$24.2 billion, depending on vaccine price scenario and speed of roll-out. In Gavi-eligible countries, the cost of vaccination would range from US$2.1 billion to US$3.8 billion, depending on the speed of roll-out. The total cost of screening and precancer treatment over this interval, which depends upon the screening scenario and speed of roll-out, would range from US$5.1 billion to either US$29.4 billion (if countries relying on Pap switched to HPV-based screening) or US$42.3 billion (if countries with existing Pap programs continued with Pap-based screening). For screening once at age 35, the cost in LI and LMI1 countries that may be dependent on foreign aid to finance screening would range from US$0.7 billion to US$1.2 billion, depending on the speed of roll-out.

In LMIC, the 10-year roll-out of HPV vaccination and cervical cancer screening programs that screen women once at age 35 had an associated cost of US$13.7 billion over the next 10 years; the average cost per girl vaccinated would be US$30.19 and the average cost per woman of screening age during this period would be US$11.87. HPV vaccination of young girls and screening of women at ages 30 and 40 years with a 10-year roll-out had an associated cost of US$18.3 billion (cost per woman of screening age: US$23.13). With more frequent, HPV-based screening, the cost per woman of screening age may reach US$38.41. A shift to HPV-based testing may provide some cost savings relative to Pap testing if screening frequency can be reduced from every 3 to every 5 years. Furthermore, HPV with VIA triage may reduce overall costs relative to HPV testing by sending fewer women to treatment.

There are several limitations to this analysis. In the absence of country-specific screening guidelines in most settings, we made simplifying assumptions regarding screening protocols and management of screen-positive women that assumed full capacity to provide treatment of precancer and did not account for the potential presence of multiple screening modalities in any given country; loss-to-follow-up of screen-positive women; varying management strategies by country; or imperfect performance of colposcopy. We focused upon screening and triage algorithms recommended by WHO guidelines [10], and thus while we considered HPV testing followed by triage with VIA, we did not consider a scenario where HPV-positive women were triaged based on Pap testing, although this is a possible path countries with existing Pap programs might consider. We assumed women were only eligible for screening at precise ages over the 10-year time horizon; thus, costs do not reflect the same frequency of screening in every woman’s lifetime, but rather the number of screenings that fall within the 10-year horizon based on the population age structure. We have not explicitly considered more frequent screening in women with HIV or in areas with high endemic HIV infection, although WHO guidelines recommend routine screening within 3 years [10]; however, we considered VIA every three years in LI and LMI1 (Scenarios 5 and 6), where the burden of HIV is greatest. In the absence of country-specific epidemiologic data in many settings, we relied upon model-based extrapolation techniques, including multivariate regression (to predict HPV prevalence) and a calibrated microsimulation model (to predict lesion prevalence); there remains uncertainty in these estimates due to potential issues with quality of epidemiologic data and microsimulation model structure. Because published and primary cost data on HPV vaccine delivery and cervical cancer screening and treatment are limited to a handful of settings, we extrapolated these cost data by using the WHO-CHOICE tool [38]; due to inconsistencies in cost reporting across the literature, we cannot be certain that the published and primary data cost estimates we used contain comparable components. We attempted to address this by considering all available data that was described in adequate detail, and we used the average of extrapolated values to account for variability and uncertainty.

This study provides much-needed cost estimates for HPV vaccination and cervical cancer screening, yet important questions remain. First, further data are needed to determine the programmatic investment costs that may be necessary to achieve high coverage levels of screening and vaccination; current data are insufficient to establish the point at which economies of scale may be counterbalanced by increasing programmatic costs of achieving high coverage. Programmatic costs of training health providers and providing quality assurance may be substantial, and will likely vary by procedure. Second, the HPV vaccine delivery costs we considered were primarily from demonstration projects; further data on the costs of delivery with national scale-up are needed. Third, the impact of future events such as patent expiration, competition from second-generation vaccines, or licensing agreements obtained by developing country vaccine manufacturers is uncertain [39]; the impact of the U.S. Food and Drug Administration’s recent approval of an HPV 9-valent vaccine on the price of first generation vaccines and prospects for generic manufacturing or voluntary licensing deals is unclear. Fourth, while we found that HPV testing with VIA triage of HPV-positive women may lead to lower total costs than HPV testing alone due to fewer women being referred to treatment, data on performance of HPV-VIA triage suggests a low sensitivity for CIN2+ [40,41], which may compromise health gains associated with screening and treatment.

In 2015, US$36.4 billion of development assistance for health was disbursed [42]. Of this, US$10.8 billion was allocated for HIV/AIDS, US$6.5 billion for child and newborn health, and US$3.6 billion for maternal health. By comparison, the present study found that the average annual cost for screening women once at age 35 would be approximately US$900 million; annual costs of HPV vaccination for all 10-year old girls (Vaccination Scenario A) would be US$1.55 billion. Decision makers will need to consider additional data on the health impact and relative cost-effectiveness of cervical cancer prevention strategies in relation to interventions for competing health priorities to determine the best value for public health dollars.

This study provides the first estimate of a price tag for comprehensive cervical cancer prevention in LMIC, including HPV vaccination of pre-adolescent girls and screening and treatment of women aged 30 to 49 years. In 2015, there were nearly 50 million 10-year-old girls and 760 million women of screening age in LMIC. For donors who fund Gavi, the Vaccine Alliance, the total cost of HPV vaccination in Gavi-eligible countries over 10 years is estimated at US$2.1 to $3.8 billion. In countries that are not eligible for Gavi assistance, the total cost of HPV vaccination is estimated at US$6.5 billion to US$20.4 billion, demonstrating the potential impact of vaccine price negotiations in middle-income countries. For those who support the health budgets of LI and LMI countries, the total cost of screening and treatment of precancer is estimated at US$0.7 to US$7.8 billion. We hope this analysis will catalyze the current policy dialogue to expediently secure necessary resources and facilitate country-level discourse on strengthening health infrastructure, identifying preferences for vaccine delivery mechanism and screening test, gathering setting-specific comparative and cost-effectiveness data, and determining timing of program introduction.

## Supporting Information

S1 File Model inputs and supplementary results. (DOCX)

## Abbreviations

CIN: cervical intraepithelial neoplasia
EAP: East Asia and Pacific region
ECA: Europe and Central Asia region
GDP: gross domestic product
GNI: gross national income
HPV: human papillomavirus
HPV-VIA: human papillomavirus testing followed by triage with visual inspection
LAC: Latin America and Caribbean region
LEEP: loop electrosurgical excision procedure
LI: low-income countries
LMI: lower-middle-income countries
LMIC: low- and middle-income countries
MENA: Middle East and North Africa region
PAHO: Pan American Health Organization
PAHO RF: Pan American Health Organization Revolving Fund
SA: South Asia region
SSA: Sub-Saharan Africa region
UMI: upper-middle-income countries
US$: United States dollars
VIA: visual inspection with acetic acid
WHO: World Health Organization
