# Supplementary material for: Resources Required for Cervical Cancer Prevention in Low- and Middle-Income Countries
Source: PLoS One. 2016 Oct 6;11(10):e0164000. doi: 10.1371/journal.pone.0164000 (PMC5053484; doi:10.1371/journal.pone.0164000)
Supplement: S1 File — (DOCX) [file pone.0164000.s001.docx]

**Appendix**

**to accompany**

**Resources Required for Cervical Cancer Prevention in Low- and Middle-Income Countries**

| **Table A. Countries included in the study, by income tier.** | | | | | |
| --- | --- | --- | --- | --- | --- |
| Count | Low Income (LI) | Lower-middle income 1 (LMI1) | Lower-middle income 2 (LMI2) | Upper-middle income 1 (UMI1) | Upper-middle income 2 (UMI2) |
|  | ≤$1045 | $1046-$2585 | $2586-$4125 | $4126-$8435 | $8436-12745 |
| 1 | Afghanistan^b^ | Bolivia | Armenia | Albania | Argentina^a^ |
| 2 | Bangladesh^b^ | Cameroon^b^ | Congo, Rep. | Algeria | Brazil |
| 3 | Benin^b^ | Cote d'Ivoire^b^ | Egypt | Angola | Costa Rica |
| 4 | Burkina Faso^b^ | Ghana^b^ | El Salvador | Azerbaijan | Gabon |
| 5 | Burundi^b^ | Honduras | Georgia | Belarus | Hungary |
| 6 | Cambodia^b^ | India^b^ | Guatemala | Bosnia and Herzegovina | Kazakhstan |
| 7 | Central African Republic^b^ | Kyrgyz Republic^b^ | Indonesia | Botswana | Lebanon |
| 8 | Chad^b^ | Lao PDR^b^ | Mongolia | Bulgaria | Malaysia |
| 9 | Congo, Dem. Rep.^b^ | Lesotho^b^ | Morocco | China | Mauritius |
| 10 | Eritrea^b^ | Mauritania^b^ | Nigeria | Colombia | Mexico |
| 11 | Ethiopia^b^ | Moldova | Paraguay | Dominican Republic | Panama |
| 12 | Gambia, The^b^ | Nicaragua | Philippines | Ecuador | Romania |
| 13 | Guinea^b^ | Pakistan^b^ | Sri Lanka | Jamaica | Turkey |
| 14 | Guinea-Bissau^b^ | Papua N. Guinea | Swaziland | Jordan | Venezuela |
| 15 | Haiti^b^ | Senegal^b^ | Timor-Leste | Macedonia, FYR |  |
| 16 | Kenya^b^ | Sudan^b^ | Ukraine | Namibia |  |
| 17 | Liberia^b^ | Uzbekistan |  | Peru |  |
| 18 | Madagascar^b^ | Vietnam^b^ |  | Serbia |  |
| 19 | Malawi^b^ | Yemen, Rep. ^b^ |  | South Africa |  |
| 20 | Mali^b^ | Zambia^b^ |  | Thailand |  |
| 21 | Mozambique^b^ |  |  | Tunisia |  |
| 22 | Nepal^b^ |  |  | Turkmenistan |  |
| 23 | Niger^b^ |  |  |  |  |
| 24 | Rwanda^b^ |  |  |  |  |
| 25 | Sierra Leone^b^ |  |  |  |  |
| 26 | Tajikistan^b^ |  |  |  |  |
| 27 | Tanzania^b^ |  |  |  |  |
| 28 | Togo^b^ |  |  |  |  |
| 29 | Uganda^b^ |  |  |  |  |
| 30 | Zimbabwe^b^ |  |  |  |  |

^a^ Argentina was classified as UMI2, although no GNI per capita data is available.

^b^ Eligible for assistance from Gavi, the Vaccine Alliance.

**Table B. Countries included in the study, by region.**

|  | Sub-Saharan Africa | East Asia & Pacific | Europe & Central Asia | Latin America & Caribbean | Middle East & North Africa | South Asia |
| --- | --- | --- | --- | --- | --- | --- |
| 1 | Angola | Cambodia | Albania | Argentina | Algeria | Afghanistan |
| 2 | Benin | China | Armenia | Bolivia | Egypt | Bangladesh |
| 3 | Botswana | Indonesia | Azerbaijan | Brazil | Jordan | India |
| 4 | Burkina Faso | Lao PDR | Belarus | Colombia | Lebanon | Nepal |
| 5 | Burundi | Malaysia | Bosnia and Herzegovina | Costa Rica | Morocco | Pakistan |
| 6 | Cameroon | Mongolia | Bulgaria | Dominican Republic | Tunisia | Sri Lanka |
| 7 | Central African Republic | Papua N. Guinea | Georgia | Ecuador | Yemen, Rep. |  |
| 8 | Chad | Philippines | Hungary | El Salvador |  |  |
| 9 | Congo, Dem. Rep. | Thailand | Kazakhstan | Guatemala |  |  |
| 10 | Congo, Rep. | Timor-Leste | Kyrgyz Republic | Haiti |  |  |
| 11 | Côte d'Ivoire | Vietnam | Macedonia, FYR | Honduras |  |  |
| 12 | Eritrea |  | Moldova | Jamaica |  |  |
| 13 | Ethiopia |  | Romania | Mexico |  |  |
| 14 | Gabon |  | Serbia | Nicaragua |  |  |
| 15 | Gambia, The |  | Tajikistan | Panama |  |  |
| 16 | Ghana |  | Turkey | Paraguay |  |  |
| 17 | Guinea |  | Turkmenistan | Peru |  |  |
| 18 | Guinea-Bissau |  | Ukraine | Venezuela |  |  |
| 19 | Kenya |  | Uzbekistan |  |  |  |
| 20 | Lesotho |  |  |  |  |  |
| 21 | Liberia |  |  |  |  |  |
| 22 | Madagascar |  |  |  |  |  |
| 23 | Malawi |  |  |  |  |  |
| 24 | Mali |  |  |  |  |  |
| 25 | Mauritania |  |  |  |  |  |
| 26 | Mauritius |  |  |  |  |  |
| 27 | Mozambique |  |  |  |  |  |
| 28 | Namibia |  |  |  |  |  |
| 29 | Niger |  |  |  |  |  |
| 30 | Nigeria |  |  |  |  |  |
| 31 | Rwanda |  |  |  |  |  |
| 32 | Senegal |  |  |  |  |  |
| 33 | Sierra Leone |  |  |  |  |  |
| 34 | South Africa |  |  |  |  |  |
| 35 | Sudan |  |  |  |  |  |
| 36 | Swaziland |  |  |  |  |  |
| 37 | Tanzania |  |  |  |  |  |
| 38 | Togo |  |  |  |  |  |
| 39 | Uganda |  |  |  |  |  |
| 40 | Zambia |  |  |  |  |  |
| 41 | Zimbabwe |  |  |  |  |  |

**Estimating HPV prevalence**

We adjusted countries with unusually high or low predicted HPV prevalence (relative to cancer burden) in the following manner. We calculated the regional HPV prevalence from countries with available data, as well as regional cancer incidence from registries and Globocan; if the difference between the model-predicted HPV prevalence and the regional HPV prevalence was more than 10%, we examined cervical cancer incidence. If country-specific cervical cancer incidence was substantially different than regional cancer incidence (e.g., the model predicted higher HPV prevalence because the country’s cervical cancer incidence was higher than regional estimates), we used model estimates. Otherwise, we substituted for the model-predicted HPV prevalence either 1) the regional HPV prevalence (if cancer incidence in the country in question was similar to regional cancer incidence); or 2) HPV prevalence survey data from a neighboring country (if cancer incidence in the country in question was similar to cancer incidence in a neighboring country). Neighboring country substitutions were made for Malawi (substitution: Mozambique) and Rwanda and Uganda (substitution: Kenya). HPV prevalence inputs are presented in **Table C in S1 File.**

**Table C. HPV prevalence inputs, by country and age group.**

| Country | Age 30-34 | Age 35-39 | Age 40-44 | Age 45-49 |
| --- | --- | --- | --- | --- |
| Afghanistan | 0.188 ^b^ | 0.229 ^b^ | 0.155 ^b^ | 0.385 ^b^ |
| Albania | 0.248 ^b^ | 0.239 ^c^ | 0.168 ^c^ | 0.105 ^b^ |
| Algeria | 0.250 ^a^ | 0.000 ^a^ | 0.111 ^a^ | 0.333 ^a^ |
| Angola | 0.383 ^b^ | 0.104 ^b^ | 0.177 ^b^ | 0.269 ^b^ |
| Argentina | 0.172 ^a^ | 0.140 ^a^ | 0.143 ^a^ | 0.118 ^a^ |
| Armenia | 0.269 ^c^ | 0.239 ^c^ | 0.168 ^c^ | 0.127 ^c^ |
| Azerbaijan | 0.236 ^b^ | 0.223 ^b^ | 0.162 ^b^ | 0.093 ^b^ |
| Bangladesh | 0.140 ^b^ | 0.127 ^b^ | 0.143 ^b^ | 0.174 ^b^ |
| Belarus | 0.245 ^b^ | 0.189 ^b^ | 0.160 ^b^ | 0.101 ^b^ |
| Benin | 0.129 ^b^ | 0.265 ^b^ | 0.136 ^b^ | 0.132 ^b^ |
| Bolivia | 0.105 ^b^ | 0.148 ^b^ | 0.141 ^b^ | 0.118 ^b^ |
| Bosnia and Herzegovina | 0.345 ^b^ | 0.203 ^b^ | 0.182 ^b^ | 0.196 ^b^ |
| Botswana | 0.316 ^b^ | 0.186 ^b^ | 0.153 ^b^ | 0.231 ^b^ |
| Brazil | 0.142 ^a^ | 0.118 ^a^ | 0.100 ^a^ | 0.114 ^a^ |
| Bulgaria | 0.385 ^b^ | 0.181 ^b^ | 0.204 ^b^ | 0.191 ^b^ |
| Burkina Faso | 0.397 ^b^ | 0.240 ^b^ | 0.273 ^b^ | 0.364 ^b^ |
| Burundi | 0.312 ^b^ | 0.140 ^b^ | 0.206 ^b^ | 0.358 ^b^ |
| Cambodia | 0.099 ^b^ | 0.112 ^b^ | 0.111 ^b^ | 0.142 ^b^ |
| Cameroon | 0.300 ^c^ | 0.282 ^c^ | 0.245 ^c^ | 0.198 ^c^ |
| Central African Republic | 0.341 ^b^ | 0.419 ^b^ | 0.294 ^b^ | 0.252 ^b^ |
| Chad | 0.300 ^c^ | 0.282 ^c^ | 0.245 ^c^ | 0.198 ^c^ |
| China | 0.098 ^a^ | 0.144 ^a^ | 0.161 ^a^ | 0.162 ^a^ |
| Colombia | 0.148 ^a^ | 0.098 ^a^ | 0.093 ^a^ | 0.077 ^a^ |
| Congo, Dem. Rep. | 0.333 ^b^ | 0.281 ^b^ | 0.288 ^b^ | 0.241 ^b^ |
| Congo, Rep. | 0.216 ^b^ | 0.282 ^c^ | 0.289 ^b^ | 0.079 ^b^ |
| Costa Rica | 0.129 ^b^ | 0.086 ^b^ | 0.100 ^b^ | 0.118 ^b^ |
| Cote d'Ivoire | 0.385 ^a^ | 0.385 ^a^ | 0.273 ^a^ | 0.182 ^a^ |
| Dominican Republic | 0.090 ^b^ | 0.042 ^b^ | 0.076 ^b^ | 0.082 ^b^ |
| Ecuador | 0.076 ^b^ | 0.055 ^b^ | 0.045 ^b^ | 0.202 ^b^ |
| Egypt | 0.037 ^a^ | 0.281 ^a^ | 0.103 ^a^ | 0.067 ^a^ |
| El Salvador | 0.170 ^c^ | 0.152 ^c^ | 0.149 ^c^ | 0.155 ^c^ |
| Eritrea | 0.343 ^b^ | 0.277 ^b^ | 0.276 ^b^ | 0.260 ^b^ |
| Ethiopia | 0.377 ^b^ | 0.229 ^b^ | 0.288 ^b^ | 0.297 ^b^ |
| Gabon | 0.224 ^c^ | 0.212 ^b^ | 0.256 ^b^ | 0.236 ^c^ |
| Gambia, The | 0.300 ^c^ | 0.282 ^c^ | 0.245 ^c^ | 0.069 ^b^ |
| Georgia | 0.269 ^c^ | 0.239 ^c^ | 0.168 ^c^ | 0.127 ^c^ |
| Ghana | 0.300 ^c^ | 0.282 ^c^ | 0.245 ^c^ | 0.198 ^c^ |
| Guatemala | 0.344 ^a^ | 0.286 ^a^ | 0.313 ^a^ | 0.273 ^a^ |
| Guinea | 0.300 ^c^ | 0.272 ^b^ | 0.245 ^c^ | 0.198 ^c^ |
| Guinea-Bissau | 0.177 ^b^ | 0.224 ^b^ | 0.160 ^b^ | 0.183 ^b^ |

**Table C (ctnd.) HPV prevalence inputs, by country and age group.**

| Country | Age 30-34 | Age 35-39 | Age 40-44 | Age 45-49 |
| --- | --- | --- | --- | --- |
| Haiti | 0.170 ^c^ | 0.152 ^c^ | 0.149 ^c^ | 0.155 ^c^ |
| Honduras | 0.361 ^a^ | 0.351 ^a^ | 0.342 ^a^ | 0.393 ^a^ |
| Hungary | 0.350 ^b^ | 0.191 ^b^ | 0.196 ^b^ | 0.158 ^b^ |
| India | 0.127 ^a^ | 0.135 ^a^ | 0.120 ^a^ | 0.126 ^a^ |
| Indonesia | 0.227 ^a^ | 0.316 ^a^ | 0.324 ^a^ | 0.178 ^a^ |
| Jamaica | 0.178 ^b^ | 0.062 ^b^ | 0.112 ^b^ | 0.172 ^b^ |
| Jordan | 0.150 ^b^ | 0.094 ^b^ | 0.086 ^b^ | 0.253 ^b^ |
| Kazakhstan | 0.319 ^b^ | 0.255 ^b^ | 0.192 ^b^ | 0.121 ^b^ |
| Kenya | 0.345 ^a^ | 0.477 ^a^ | 0.274 ^a^ | 0.348 ^a^ |
| Kyrgyz Republic | 0.313 ^b^ | 0.537 ^b^ | 0.290 ^b^ | 0.169 ^b^ |
| Lao PDR | 0.146 ^b^ | 0.229 ^b^ | 0.173 ^b^ | 0.118 ^b^ |
| Lebanon | 0.138 ^b^ | 0.086 ^b^ | 0.080 ^b^ | 0.239 ^b^ |
| Lesotho | 0.301 ^b^ | 0.192 ^b^ | 0.247 ^b^ | 0.195 ^b^ |
| Liberia | 0.177 ^b^ | 0.306 ^b^ | 0.177 ^b^ | 0.156 ^b^ |
| Macedonia, FYR | 0.264 ^b^ | 0.167 ^b^ | 0.161 ^b^ | 0.125 ^b^ |
| Madagascar | 0.311 ^b^ | 0.304 ^b^ | 0.251 ^b^ | 0.247 ^b^ |
| Malawi | 0.414 ^d^ | 0.263 ^d^ | 0.333 ^d^ | 0.200 ^d^ |
| Malaysia | 0.100 ^b^ | 0.069 ^b^ | 0.078 ^b^ | 0.086 ^b^ |
| Mali | 0.381 ^b^ | 0.160 ^b^ | 0.264 ^b^ | 0.363 ^b^ |
| Mauritania | 0.228 ^b^ | 0.257 ^b^ | 0.209 ^b^ | 0.165 ^b^ |
| Mauritius | 0.273 ^b^ | 0.196 ^b^ | 0.152 ^b^ | 0.176 ^b^ |
| Mexico | 0.044 ^a^ | 0.060 ^a^ | 0.044 ^a^ | 0.095 ^a^ |
| Moldova | 0.285 ^b^ | 0.239 ^c^ | 0.295 ^b^ | 0.114 ^b^ |
| Mongolia | 0.257 ^a^ | 0.250 ^a^ | 0.213 ^a^ | 0.202 ^a^ |
| Morocco | 0.094 ^a^ | 0.226 ^a^ | 0.154 ^a^ | 0.227 ^a^ |
| Mozambique | 0.414 ^a^ | 0.263 ^a^ | 0.333 ^a^ | 0.200 ^a^ |
| Namibia | 0.300 ^b^ | 0.178 ^c^ | 0.202 ^c^ | 0.285 ^c^ |
| Nepal | 0.187 ^b^ | 0.139 ^b^ | 0.175 ^b^ | 0.215 ^b^ |
| Nicaragua | 0.245 ^b^ | 0.241 ^b^ | 0.245 ^b^ | 0.213 ^b^ |
| Niger | 0.300 ^c^ | 0.282 ^c^ | 0.245 ^c^ | 0.292 ^b^ |
| Nigeria | 0.224 ^a^ | 0.258 ^a^ | 0.254 ^a^ | 0.236 ^a^ |
| Pakistan | 0.192 ^b^ | 0.199 ^b^ | 0.172 ^b^ | 0.304 ^b^ |
| Panama | 0.152 ^b^ | 0.090 ^b^ | 0.107 ^b^ | 0.143 ^b^ |
| Papua N. Guinea | 0.253 ^b^ | 0.198 ^b^ | 0.205 ^b^ | 0.256 ^b^ |
| Paraguay | 0.182 ^a^ | 0.176 ^a^ | 0.167 ^a^ | 0.167 ^a^ |
| Peru | 0.048 ^a^ | 0.054 ^a^ | 0.086 ^a^ | 0.085 ^a^ |
| Philippines | 0.075 ^a^ | 0.143 ^a^ | 0.094 ^a^ | 0.071 ^a^ |
| Romania | 0.385 ^b^ | 0.235 ^b^ | 0.202 ^b^ | 0.225 ^b^ |
| Rwanda | 0.345 ^d^ | 0.477 ^d^ | 0.274 ^d^ | 0.348 ^d^ |
| Senegal | 0.111 ^a^ | 0.113 ^a^ | 0.139 ^a^ | 0.092 ^a^ |

**Table C (ctnd.) HPV prevalence inputs, by country and age group.**

| Country | Age 30-34 | Age 35-39 | Age 40-44 | Age 45-49 |
| --- | --- | --- | --- | --- |
| Serbia | 0.410 ^b^ | 0.210 ^b^ | 0.213 ^b^ | 0.215 ^b^ |
| Sierra Leone | 0.185 ^b^ | 0.324 ^b^ | 0.184 ^b^ | 0.161 ^b^ |
| South Africa | 0.322 ^a^ | 0.195 ^a^ | 0.199 ^a^ | 0.130 ^a^ |
| Sri Lanka | 0.097 ^b^ | 0.187 ^b^ | 0.128 ^b^ | 0.096 ^b^ |
| Sudan | 0.355 ^b^ | 0.388 ^b^ | 0.312 ^b^ | 0.197 ^b^ |
| Swaziland | 0.288 ^b^ | 0.282 ^c^ | 0.245 ^c^ | 0.261 ^b^ |
| Tajikistan | 0.278 ^b^ | 0.239 ^c^ | 0.168 ^c^ | 0.127 ^c^ |
| Tanzania | 0.300 ^a^ | 0.282 ^a^ | 0.245 ^a^ | 0.203 ^a^ |
| Thailand | 0.118 ^b^ | 0.060 ^b^ | 0.049 ^b^ | 0.056 ^b^ |
| Timor-Leste | 0.089 ^b^ | 0.153 ^b^ | 0.118 ^b^ | 0.089 ^b^ |
| Togo | 0.237 ^b^ | 0.303 ^b^ | 0.207 ^b^ | 0.207 ^b^ |
| Tunisia | 0.167 ^a^ | 0.105 ^a^ | 0.074 ^a^ | 0.333 ^a^ |
| Turkey | 0.147 ^b^ | 0.096 ^b^ | 0.084 ^b^ | 0.254 ^b^ |
| Turkmenistan | 0.203 ^b^ | 0.162 ^b^ | 0.140 ^b^ | 0.082 ^b^ |
| Uganda | 0.345 ^d^ | 0.228 ^b^ | 0.318 ^b^ | 0.348 ^d^ |
| Ukraine | 0.269 ^c^ | 0.239 ^c^ | 0.168 ^c^ | 0.127 ^c^ |
| Uzbekistan | 0.281 ^b^ | 0.478 ^b^ | 0.296 ^b^ | 0.112 ^b^ |
| Venezuela | 0.216 ^b^ | 0.089 ^b^ | 0.133 ^b^ | 0.176 ^b^ |
| Vietnam | 0.045 ^a^ | 0.049 ^a^ | 0.034 ^a^ | 0.019 ^a^ |
| Yemen, Rep. | 0.136 ^b^ | 0.218 ^b^ | 0.142 ^b^ | 0.229 ^b^ |
| Zambia | 0.300 ^c^ | 0.282 ^c^ | 0.245 ^c^ | 0.198 ^c^ |
| Zimbabwe | 0.300 ^c^ | 0.282 ^c^ | 0.245 ^c^ | 0.198 ^c^ |

^a^ Prevalence estimate from survey data.

^b^ Prevalence estimate from predictive model. R^2^ values: 0.64 (Age 30-34); 0.70 (Age 35-39); 0.59 (Age 40-44); 0.42 (Age 45-49).

^c^ Prevalence estimate from regional survey data.

^d^ Prevalence estimate from neighboring country with similar cancer incidence (Malawi: used Mozambique prevalence estimates; Rwanda: used Kenya prevalence estimates; Uganda: used Kenya prevalence estimates).

**Table D. Cervical cancer incidence inputs, by country and age group [**[**1**](#_ENREF_1)**].**

| Country | Age 30-34 | Age 35-39 | Age 40-44 | Age 45-49 |
| --- | --- | --- | --- | --- |
| Afghanistan | 9.3 | 15.3 | 21.9 | 27.0 |
| Albania | 5.9 | 12.5 | 15.3 | 9.8 |
| Algeria | 1.0 | 4.0 | 9.9 | 18.4 |
| Angola | 28.5 | 44.0 | 61.3 | 78.8 |
| Argentina | 26.7 | 37.4 | 44.2 | 46.3 |
| Armenia | 28.2 | 42.5 | 47.0 | 40.0 |
| Azerbaijan | 13.7 | 18.5 | 21.5 | 23.2 |
| Bangladesh | 9.6 | 20.4 | 35.9 | 54.9 |
| Belarus | 16.2 | 22.2 | 27.3 | 30.2 |
| Benin | 15.2 | 25.5 | 37.4 | 48.2 |
| Bolivia | 43.1 | 65.9 | 87.9 | 104.8 |
| Bosnia and Herzegovina | 14.4 | 29.1 | 33.3 | 40.2 |
| Botswana | 30.7 | 47.0 | 55.4 | 60.5 |
| Brazil | 15.4 | 21.8 | 27.1 | 31.6 |
| Bulgaria | 30.0 | 43.3 | 55.4 | 60.4 |
| Burkina Faso | 13.1 | 24.4 | 39.9 | 57.1 |
| Burundi | 29.3 | 53.4 | 83.9 | 115.9 |
| Cambodia | 13.2 | 25.5 | 41.4 | 61.2 |
| Cameroon | 17.7 | 38.6 | 65.5 | 81.0 |
| Central African Republic | 6.9 | 16.7 | 31.7 | 44.9 |
| Chad | 9.0 | 18.6 | 32.2 | 43.7 |
| China | 10.4 | 14.3 | 17.5 | 18.2 |
| Colombia | 18.9 | 27.6 | 33.6 | 35.3 |
| Congo, Dem. Rep. | 11.5 | 25.7 | 45.7 | 74.5 |
| Congo, Rep. | 1.3 | 8.0 | 21.2 | 40.3 |
| Costa Rica | 15.3 | 20.8 | 23.9 | 25.6 |
| Cote d'Ivoire | 12.5 | 21.5 | 32.1 | 43.0 |
| Dominican Republic | 31.9 | 41.7 | 51.0 | 59.9 |
| Ecuador | 22.3 | 36.3 | 52.0 | 60.8 |
| Egypt | 1.0 | 1.8 | 3.3 | 5.2 |
| El Salvador | 47.6 | 62.1 | 63.7 | 57.0 |
| Eritrea | 6.8 | 13.0 | 22.3 | 38.6 |
| Ethiopia | 11.4 | 22.1 | 37.4 | 63.2 |
| Gabon | 14.3 | 25.5 | 38.1 | 48.5 |
| Gambia, The | 11.8 | 21.6 | 25.1 | 37.7 |
| Georgia | 19.5 | 31.6 | 38.7 | 40.6 |
| Ghana | 20.7 | 34.2 | 49.4 | 62.7 |
| Guatemala | 38.7 | 53.8 | 62.2 | 60.3 |
| Guinea | 18.5 | 33.8 | 52.1 | 68.3 |
| Guinea-Bissau | 13.7 | 26.8 | 42.2 | 60.5 |

**Table D (ctnd.) Cervical cancer incidence inputs, by country and age group [**[**1**](#_ENREF_1)**].**

| Country | Age 30-34 | Age 35-39 | Age 40-44 | Age 45-49 |
| --- | --- | --- | --- | --- |
| Haiti | 26.3 | 36.4 | 38.1 | 37.9 |
| Honduras | 47.7 | 65.3 | 73.1 | 72.6 |
| Hungary | 24.9 | 34.5 | 42.2 | 44.6 |
| India | 12.7 | 25.0 | 41.9 | 60.4 |
| Indonesia | 7.2 | 16.5 | 30.4 | 43.9 |
| Jamaica | 33.0 | 45.4 | 55.2 | 64.5 |
| Jordan | 1.3 | 3.0 | 5.0 | 8.7 |
| Kazakhstan | 49.9 | 63.0 | 64.1 | 59.6 |
| Kenya | 21.5 | 41.5 | 63.5 | 84.0 |
| Kyrgyz Republic | 38.3 | 51.4 | 57.0 | 56.0 |
| Lao PDR | 11.2 | 18.3 | 26.9 | 33.3 |
| Lebanon | 3.3 | 5.3 | 7.6 | 10.5 |
| Lesotho | 38.6 | 40.4 | 67.0 | 92.6 |
| Liberia | 15.5 | 27.3 | 42.3 | 55.3 |
| Macedonia, FYR | 10.0 | 17.3 | 26.2 | 34.9 |
| Madagascar | 38.5 | 59.5 | 71.5 | 90.9 |
| Malawi | 84.3 | 128.8 | 171.7 | 189.2 |
| Malaysia | 9.6 | 16.8 | 25.9 | 35.4 |
| Mali | 20.4 | 41.1 | 72.5 | 107.2 |
| Mauritania | 13.2 | 25.4 | 40.8 | 58.7 |
| Mauritius | 7.4 | 13.6 | 20.7 | 26.0 |
| Mexico | 26.8 | 38.5 | 46.7 | 51.1 |
| Moldova | 24.8 | 34.4 | 42.0 | 45.2 |
| Mongolia | 17.8 | 32.1 | 47.5 | 62.3 |
| Morocco | 7.3 | 14.7 | 23.3 | 33.4 |
| Mozambique | 71.4 | 90.1 | 108.2 | 132.5 |
| Namibia | 11.8 | 18.5 | 27.6 | 33.8 |
| Nepal | 8.5 | 22.9 | 36.6 | 53.3 |
| Nicaragua | 46.8 | 66.7 | 78.7 | 85.4 |
| Niger | 7.2 | 11.7 | 17.1 | 18.1 |
| Nigeria | 8.9 | 21.2 | 39.7 | 61.3 |
| Pakistan | 4.8 | 9.2 | 15.0 | 23.4 |
| Panama | 27.9 | 36.7 | 41.9 | 42.3 |
| Papua N. Guinea | 37.5 | 59.5 | 80.1 | 87.8 |
| Paraguay | 49.0 | 66.1 | 75.7 | 76.7 |
| Peru | 26.6 | 44.9 | 63.3 | 74.5 |
| Philippines | 19.0 | 28.0 | 36.5 | 40.1 |
| Romania | 25.2 | 40.2 | 56.0 | 72.1 |
| Rwanda | 22.3 | 42.0 | 68.1 | 104.9 |
| Senegal | 20.3 | 38.2 | 61.9 | 88.0 |

**Table D (ctnd.) Cervical cancer incidence inputs, by country and age group [**[**1**](#_ENREF_1)**].**

| Country | Age 30-34 | Age 35-39 | Age 40-44 | Age 45-49 |
| --- | --- | --- | --- | --- |
| Serbia | 32.0 | 45.8 | 56.8 | 63.1 |
| Sierra Leone | 15.3 | 27.5 | 42.0 | 55.4 |
| South Africa | 37.6 | 53.1 | 63.4 | 68.3 |
| Sri Lanka | 0.0 | 11.2 | 22.5 | 33.2 |
| Sudan | 2.1 | 3.7 | 6.7 | 13.5 |
| Swaziland | 59.8 | 61.8 | 101.2 | 124.1 |
| Tajikistan | 13.8 | 21.5 | 28.8 | 30.4 |
| Tanzania | 27.5 | 51.4 | 80.3 | 116.5 |
| Thailand | 15.0 | 24.7 | 36.3 | 44.5 |
| Timor-Leste | 21.3 | 8.8 | 18.2 | 35.3 |
| Togo | 10.6 | 19.2 | 30.3 | 42.5 |
| Tunisia | 1.5 | 3.1 | 5.9 | 9.6 |
| Turkey | 3.5 | 6.1 | 8.4 | 10.5 |
| Turkmenistan | 14.1 | 19.4 | 24.8 | 29.8 |
| Uganda | 30.8 | 55.2 | 87.3 | 115.5 |
| Ukraine | 23.6 | 31.6 | 37.8 | 39.2 |
| Uzbekistan | 17.6 | 24.8 | 30.7 | 34.6 |
| Venezuela | 52.0 | 66.7 | 71.5 | 70.2 |
| Vietnam | 9.4 | 14.5 | 20.5 | 26.7 |
| Yemen, Rep. | 0.6 | 2.1 | 4.3 | 7.6 |
| Zambia | 60.3 | 88.1 | 110.4 | 127.0 |
| Zimbabwe | 19.9 | 41.4 | 71.2 | 105.8 |

**Table E. Screening test performance inputs.**

| **Screening test (references)** | **True positive rate (sensitivity)^a^** | **False positive rate (1-specificity)**^b^ |
| --- | --- | --- |
| VIA [[2](#_ENREF_2),[3](#_ENREF_3)] | CIN1: 42%  CIN2/3: 60% | 16% |
| HPV [[4-8](#_ENREF_4)] | CIN1: 80%  CIN2/3: 90% |  |
| Pap [[2](#_ENREF_2),[3](#_ENREF_3)] | CIN1: 35%  CIN2/3: 60% | 9% |
| HPV-VIA^c^ [[5-10](#_ENREF_5)] | CIN1: 34%  CIN2/3: 54% |  |

^a^ With each screening test, women with CIN1 may be more likely to screen positive than women with no lesions, although they are less likely to screen positive than women with CIN2/3. We thus input separate test sensitivity values for CIN1 and CIN2/3.

^b^ For VIA and Pap, test specificity was used to derive the false positive rate associated with each screening test for purposes of establishing the number of women with no lesion who screen positive and thus may accrue further diagnostic and/or treatment costs. For HPV testing, the mechanics of the test are based on the presence or absence of HPV, and we assumed the test detected clinically relevant oncogenic HPV infections with perfect accuracy; we considered a false positive result to occur among women with HPV infection but no CIN. Because we assumed 80% of CIN1 were attributable to oncogenic HPV, and 90% of CIN2/3 were attributable to oncogenic HPV [[5-8](#_ENREF_5)], we multiplied the prevalence of CIN1 and CIN2/3 in a given country by 80% and 90% (respectively) and subtracted these values from the prevalence of oncogenic HPV in that country to generate the proportion of women with HPV who screen positive but have no lesion. Thus, the false positive rate varies by country.

^c^ Limited data on the performance of HPV testing with VIA triage suggests that sensitivity is similar to VIA alone [[9](#_ENREF_9),[10](#_ENREF_10)], so we applied VIA performance characteristics to the proportion of women with CIN attributable to oncogenic HPV.

**Table F. Attenuation factors to capture reduction in CIN1 and CIN2/3 prevalence associated with repeated screening, by screening modality and frequency.^a^**

| **Strategy** | **CIN1 Attenuation Factor** | **CIN2/3 Attenuation Factor** |
| --- | --- | --- |
| VIA Q10 | 1 | 0.87 |
| VIA Q5 | 0.97 | 0.62 |
| VIA Q3 | 0.92 | 0.46 |
| Pap Q10 | 1 | 0.85 |
| Pap Q5 | 1 | 0.58 |
| Pap Q3 | 0.96 | 0.42 |
| HPV Q10 | 1 | 0.79 |
| HPV Q5 | 0.95 | 0.49 |
| HPV-VIA Q10^b^ | 1 | 0.87 |
| HPV-VIA Q5^b^ | 0.97 | 0.62 |

^a^ Q10: screening at 10 year intervals (either at age 30, 40 years); Q5: screening at 5 year intervals (at age 30, 35, 40, 45 years); Q3: screening at 3 year intervals (at age 30, 33, 36, 39, 42, 45, 48 years).

^b^ HPV-VIA was assumed to perform similarly to VIA at the same screening frequency.

**Table G. Published HPV vaccine delivery cost per dose estimates (2013 US$).**^a^

| **Country and vaccine delivery strategy** | **HPV vaccine delivery cost per dose** |
| --- | --- |
| Tanzania (school-based) [[11](#_ENREF_11)] | 3.68 |
| Peru (school-based) ^b^ [[12](#_ENREF_12)] | 2.68 |
| Uganda (school-based) ^b^ [[12](#_ENREF_12)] | 2.44 |
| Uganda (integrated outreach) ^b^ [[12](#_ENREF_12)] | 0.95 |
| Vietnam (school-based) ^b^ [[12](#_ENREF_12)] | 0.98 |
| Vietnam (health center) ^b^ [[12](#_ENREF_12)] | 0.85 |
| Tanzania (school-based)[[13](#_ENREF_13)] | 3.19 |

^a^ Costs represent economic costs of HPV vaccine delivery, excluding the price of the vaccine.

^b^ Recurrent costs only; start-up costs have been excluded.

**Table H. Screening, diagnosis, and treatment of CIN: Procedures and location of service delivery.**

| **Procedure** | **Location of Service Delivery** |
| --- | --- |
| VIA test | Primary outpatient clinic |
| Cytology test | Primary outpatient clinic |
| HPV test | Primary outpatient clinic |
| Colposcopy/biopsy | Secondary outpatient hospital |
| Cryotherapy | Primary outpatient clinic |
| LEEP | Secondary outpatient hospital |

**Table I. Primary data costs, by procedure (2013 US$).**

| **Country** | **VIA** | **Pap** | **HPV Test^a^** | **Cryotherapy** | **Colposcopy/biopsy** | **LEEP** |
| --- | --- | --- | --- | --- | --- | --- |
| El Salvador [[14](#_ENREF_14)] | 1.95 | 4.31 | 6.90 | 22.60 | 86.64 | 45.07 |
| Ghana [[15](#_ENREF_15)] | 8.06 |  |  | 37.37 |  |  |
| India [[16](#_ENREF_16)] | 0.68 | 1.49 | 6.92 | 5.34 | 13.46 | 35.73 |
| India (Hyderabad) [[2](#_ENREF_2),[17](#_ENREF_17)] | 1.07 | 4.66 | 6.27 | 13.13 | 9.60 |  |
| India (New Delhi) [[2](#_ENREF_2),[17](#_ENREF_17)] | 1.43 | 6.38 | 6.74 | 18.12 | 15.22 |  |
| Kenya [[16](#_ENREF_16)] | 1.32 | 2.81 | 8.98 | 14.20 | 15.83 | 127.02 |
| Nicaragua [[2](#_ENREF_2),[17](#_ENREF_17)] | 4.04 | 5.94 | 9.57 | 14.60 | 19.25 | 66.15 |
| Peru [[16](#_ENREF_16)] | 3.03 | 4.72 | 10.72 | 8.62 | 7.65 | 163.18 |
| South Africa [[16](#_ENREF_16)] | 9.77 | 13.51 | 15.92 | 61.11 | 74.18 | 260.39 |
| Thailand [[16](#_ENREF_16)] | 1.09 | 2.17 | 8.07 | 21.20 | 42.39 | 169.56 |
| Uganda [[2](#_ENREF_2),[17](#_ENREF_17)] | 1.29 | 5.36 | 6.84 | 5.92 | 16.13 | 79.37 |

^a^ In converting primary data costs to 2013 US$, we assumed the HPV test has a standardized tradable value of $5 (2013 US$).

**Appendix Table J. Average HPV vaccine delivery cost per dose, by income tier and Gavi eligibility status (2013 US$).**^a^

| **Income tier** | **HPV vaccine delivery cost per dose** |
| --- | --- |
| LI ^b^ | 1.60 |
| LMI1 | 3.51 |
| LMI2 | 6.64 |
| UMI1 | 11.90 |
| UMI2^c^ | 19.43 |
|  |  |
| **Gavi-eligible** |  |
| Yes ^b^ | 2.14 |
| No ^c^ | 11.80 |

^a^ Costs represent economic costs of HPV vaccine delivery, excluding the price of the vaccine. Gavi: Gavi, the Vaccine Alliance; LI: Low Income; LMI1: Lower-middle income 1; LMI2: Lower-middle income 2; UMI1: Upper-middle income 1; UMI2: Upper-middle income 2.

In countries for which the official exchange rate for 2013 was unavailable, we used the DEC alternative conversion rate [[18](#_ENREF_18)]. Because 2013 GDP deflators were not available to convert WHO-CHOICE 2008 local currency unit costs to 2013 US$ in several countries, we used the 2012 GDP deflator [[18](#_ENREF_18)].

^b^ For Zimbabwe, we substituted cost data from Kenya as a proxy country, given the similarity in 2013 GNI per capita between Kenya and Zimbabwe. In Zambia in 2013, 1000 ZMK became equivalent to 1 ZMW, so we divided the official exchange rate by 1000.

^c^ For Brazil, WHO-CHOICE data from 2008 suggested low procedure costs that did not fit the generally linear relationship with GNI per capita, so instead of using the average extrapolated value we used the maximum extrapolated value implied by the primary data.

**Table K. Average procedure cost, by income tier (2013 US$).** ^a^

| **Income tier** | **VIA** | **Pap** | **HPV Test**^b^ | **Cryotherapy** | **Colposcopy/biopsy** | **LEEP** |
| --- | --- | --- | --- | --- | --- | --- |
| LI^c^ | 1.60 | NA | 6.61 | 11.39 | 22.74 | 47.72 |
| LMI1 | 3.52 | NA | 8.52 | 24.99 | 49.87 | 101.64 |
| LMI2 | 6.65 | 11.81 | 11.66 | 50.77 | 94.30 | 197.87 |
| UMI1 | 11.92 | 21.17 | 16.94 | 84.72 | 169.07 | 354.76 |
| UMI2^d^ | 19.46 | 34.12 | 24.11 | 137.83 | 285.17 | 565.75 |

^a^ LI: Low Income; LMI1: Lower-middle income 1; LMI2: Lower-middle income 2; UMI1: Upper-middle income 1; UMI2: Upper-middle income 2; NA: Not applicable, as these strategies were not considered for Low Income countries. In countries for which the official exchange rate for 2013 was unavailable, we used the DEC alternative conversion rate [[18](#_ENREF_18)]. Because 2013 GDP deflators were not available to convert WHO-CHOICE 2008 local currency unit costs to 2013 US$ in several countries, we used the 2012 GDP deflator [[18](#_ENREF_18)].

^b^ We assumed that the HPV test had a standardized tradable value of US$5, and did not apply the WHO-CHOICE facility ratios to this component of HPV screening costs.

^c^ For Zimbabwe, we substituted cost data from Kenya as a proxy country, given the similarity in 2013 GNI per capita between Kenya and Zimbabwe. In Zambia in 2013, 1000 ZMK became equivalent to 1 ZMW, so we divided the official exchange rate by 1000.

^d^ For Brazil, WHO-CHOICE data from 2008 suggested low procedure costs that did not fit the generally linear relationship with GNI per capita, so instead of using the average extrapolated value we used the maximum extrapolated value implied by the primary data.

**Table L. Total discounted cost of HPV vaccination from 2015 to 2024, by income tier, World Bank region, and vaccination scenario (2013 US$, billions).**^a^

| **Income tier or World Bank region** | **Number of 10-year-old girls in 2015, LMIC (% of total)** | **Vaccination scenario** | | |
| --- | --- | --- | --- | --- |
|  |  | **A** | **B** | **C** |
| ***Immediate roll-out***^b^**,** **cost (% of total costs)** | | | | |
| **TOTAL** | 49,743,665 | **$13.6 B** | **$20.6 B** | **$21.2 B** |
| ***By Income Tier*** | |  |  |  |
| **LI** | 9,868,831 (20%) | $1.1 B (8) | $1.1 B (5) | $1.1 B (5) |
| **LMI1** | 16,988,122 (34%) | $2.3 B (17) | $2.4 B (12) | $2.4 B (11) |
| **LMI2** | 7,610,480 (15%) | $2.9 B (22) | $4.8 B (23) | $4.8 B (23) |
| **UMI1** | 10,427,821 (21%) | $4.9 B (36) | $9.3 B (45) | $9.3 B (44) |
| **UMI2** | 4,848,411 (10%) | $2.3 B (17) | $2.9 B (14) | $3.6 B (17) |
| ***By World Region*** | |  |  |  |
| **EAP** | 12,545,391 (25%) | $5 B (37) | $9.8 B (48) | $10 B (47) |
| **ECA** | 1,848,914 (4%) | $0.9 B (6) | $1.5 B (8) | $2 B (10) |
| **LAC** | 5,162,675 (10%) | $2.4 B (17) | $2.4 B (12) | $2.4 B (11) |
| **MENA** | 1,909,780 (4%) | $0.7 B (5) | $1.3 B (6) | $1.3 B (6) |
| **SA** | 15,897,310 (32%) | $2.1 B (15) | $2.1 B (10) | $2.1 B (10) |
| **SSA** | 12,379,595 (25%) | $2.5 B (19) | $3.4 B (16) | $3.4 B (16) |
| ***By Gavi eligibility*** | |  |  |  |
| **Gavi-eligible^c^** | 26,216,170 (53%) | $3.3 B (24) | $3.3 B (16) | $3.3 B (16) |
| **Non-Gavi eligible** | 23,527,495 (47%) | $10.3 B (76) | $17.2 B (84) | $17.9 B (84) |
|  |  |  |  |  |
| ***5-year roll-out***^b^, **cost (% of total costs)** | | | | |
| **TOTAL** | 49,743,665 | **$10.7 B** | **$16.2 B** | **$16.7 B** |
| ***By Income Tier*** | |  |  |  |
| **LI** | 9,868,831 (20%) | $0.9 B (8) | $0.9 B (5) | $0.9 B (5) |
| **LMI1** | 16,988,122 (34%) | $1.8 B (17) | $1.9 B (12) | $1.9 B (11) |
| **LMI2** | 7,610,480 (15%) | $2.3 B (22) | $3.8 B (24) | $3.8 B (23) |
| **UMI1** | 10,427,821 (21%) | $3.9 B (36) | $7.3 B (45) | $7.3 B (44) |
| **UMI2** | 4,848,411 (10%) | $1.8 B (17) | $2.3 B (14) | $2.8 B (17) |
| ***By World Region*** | |  |  |  |
| **EAP** | 12,545,391 (25%) | $4 B (37) | $7.7 B (48) | $7.8 B (47) |
| **ECA** | 1,848,914 (4%) | $0.7 B (6) | $1.2 B (7) | $1.6 B (10) |
| **LAC** | 5,162,675 (10%) | $1.8 B (17) | $1.9 B (12) | $1.9 B (11) |
| **MENA** | 1,909,780 (4%) | $0.6 B (5) | $1 B (6) | $1 B (6) |
| **SA** | 15,897,310 (32%) | $1.6 B (15) | $1.6 B (10) | $1.6 B (10) |
| **SSA** | 12,379,595 (25%) | $2 B (19) | $2.7 B (17) | $2.7 B (16) |
| ***By Gavi eligibility*** | |  |  |  |
| **Gavi-eligible^c^** | 26,216,170 (53%) | $2.6 B (24) | $2.6 B (16) | $2.6 B (16) |
| **Non-Gavi eligible** | 23,527,495 (47%) | $8.1 B (76) | $13.6 B (84) | $14.1 B (84) |
|  |  |  |  |  |
| ***10-year roll-out***^b^, **cost (% of total costs)** | | | | |
| **TOTAL** | 49,743,665 | **$7.2 B** | **$11 B** | **$11.3 B** |
| ***By Income Tier*** | |  |  |  |
| **LI** | 9,868,831 (20%) | $0.6 B (8) | $0.6 B (5) | $0.6 B (5) |
| **LMI1** | 16,988,122 (34%) | $1.2 B (17) | $1.3 B (12) | $1.3 B (11) |
| **LMI2** | 7,610,480 (15%) | $1.6 B (22) | $2.6 B (24) | $2.6 B (23) |
| **UMI1** | 10,427,821 (21%) | $2.6 B (36) | $5 B (45) | $5 B (44) |
| **UMI2** | 4,848,411 (10%) | $1.2 B (17) | $1.5 B (14) | $1.9 B (16) |
| ***By World Region*** | |  |  |  |
| **EAP** | 12,545,391 (25%) | $2.7 B (37) | $5.3 B (48) | $5.3 B (47) |
| **ECA** | 1,848,914 (4%) | $0.5 B (6) | $0.8 B (7) | $1.1 B (9) |
| **LAC** | 5,162,675 (10%) | $1.2 B (17) | $1.3 B (12) | $1.3 B (11) |
| **MENA** | 1,909,780 (4%) | $0.4 B (5) | $0.7 B (6) | $0.7 B (6) |
| **SA** | 15,897,310 (32%) | $1.1 B (15) | $1.1 B (10) | $1.1 B (10) |
| **SSA** | 12,379,595 (25%) | $1.4 B (19) | $1.8 B (17) | $1.9 B (16) |
| ***By Gavi eligibility*** | |  |  |  |
| **Gavi-eligible^c^** | 26,216,170 (53%) | $1.8 B (24) | $1.8 B (16) | $1.8 B (16) |
| **Non-Gavi eligible** | 23,527,495 (47%) | $5.5 B (76) | $9.2 B (84) | $9.6 B (84) |

^a^ Gavi: Gavi, the Vaccine Alliance; LMIC: low- and middle-income countries; LI: Low Income; LMI1: Lower-middle income tier 1; LMI2: Lower-middle income tier 2; UMI1: Upper-middle income tier 1; UMI2: Upper-middle income tier 2; EAP: East Asia & Pacific; ECA: Europe & Central Asia; LAC: Latin America & Caribbean; MENA: Middle East & North Africa; SA: South Asia; SSA: Sub-Saharan Africa.

^b^ Immediate roll-out: Full coverage (100%) of the target population from 2015 to 2024; 5-year roll-out: 20% coverage in 2015, 40% coverage in 2016, 60% coverage in 2017, 80% coverage in 2018, and 100% coverage of the target population from 2019 to 2024; 10-year roll-out: 10% coverage in 2015, 20% coverage in 2016, 30% coverage in 2017, 40% coverage in 2018, 50% coverage in 2019, 60% coverage in 2020, 70% coverage in 2021, 80% coverage in 2022, 90% coverage in 2023, and 100% coverage of the target population in 2024.

**Table M. Total discounted cost of cervical cancer screening from 2015 to 2024, by income tier, World Bank region, and screening scenario (2013 US$, billions).**^a^

| **Income tier or World Bank region** | **Number of women aged 30-49 years in 2015, LMIC (% of total)** | **Screening scenario** | | | | | | |
| --- | --- | --- | --- | --- | --- | --- | --- | --- |
|  |  | **1** | **2** | **3** | **4** | **5** | **6** | **7** |
| ***Immediate roll-out***^b^**, *cost (% of total costs)*** | | | | | | | | |
| **TOTAL** | 760,402,598 | **$7.7 B** | **$15.0 B** | **$31.2 B** | **$26.5 B** | **$36.0 B** | **$25.0 B** | **$24.9 B** |
| ***By Income Tier*** | | | | | | | | |
| **LI** | 84,739,921 (11%) | $0.2 B (3) | $0.4 B (3) | $0.7 B (2) | $0.7 B (3) | $1.2 B (3) | $1.2 B (5) | $1.2 B (5) |
| **LMI1** | 230,936,378 (30%) | $0.9 B (11) | $1.7 B (12) | $3.3 B (10) | $3.3 B (12) | $5.5 B (15) | $5.5 B (22) | $5.3 B (21) |
| **LMI2** | 94,895,600 (12%) | $0.9 B (12) | $1.7 B (12) | $5.2 B (17) | $5.1 B (20) | $4.8 B (13) | $4.4 B (18) | $4.4 B (18) |
| **UMI1** | 263,137,970 (35%) | $3.5 B (45) | $6.8 B (45) | $13.7 B (44) | $13.0 B (50) | $11.5 B (32) | $9.6 B (38) | $9.6 B (39) |
| **UMI2** | 86,992,729 (11%) | $2.2 B (28) | $4.3 B (29) | $8.4 B (26) | $4.4 B (16) | $13.0 B (36) | $4.4 B (18) | $4.4 B (18) |
| ***By World Region*** | | | | | | | | |
| **EAP** | 295,355,455 (39%) | $3.2 B (41) | $5.9 B (39) | $13.2 B (42) | $13.2 B (50) | $10.4 B (29) | $10.4 B (42) | $10.3 B (41) |
| **ECA** | 39,367,836 (5%) | $0.7 B (9) | $1.3 B (9) | $2.6 B (8) | $2.2 B (8) | $3.3 B (9) | $2.1 B (8) | $2.1 B (9) |
| **LAC** | 82,566,777 (11%) | $2.2 B (29) | $4.4 B (30) | $8.5 B (27) | $4.2 B (16) | $13.9 B (39) | $4.1 B (16) | $4.0 B (16) |
| **MENA** | 27,105,360 (4%) | $0.2 B (3) | $0.5 B (4) | $ 1.3 B (4) | $1.3 B (5) | $1.0 B (3) | $1.0 B (4) | $1.0 B (4) |
| **SA** | 220,923,612 (29%) | $0.8 B (10) | $1.5 B (10) | $3.0 B (9) | $3.0 B (11) | $4.7 B (13) | $4.7 B (19) | $4.6 B (18) |
| **SSA** | 95,083,558 (13%) | $0.6 B (8) | $1.3 B (8) | $2.6 B (8) | $2.6 B (10) | $2.7 B (7) | $2.7 B (11) | $2.8 B (11) |
| ***5-year roll-out***^b^**, *cost (% of total costs)*** | | | | | | | | |
| **TOTAL** | 760,402,598 | **$6.1 B** | **$11.7 B** | **$24.4 B** | **$20.8 B** | **$28.2 B** | **$19.6 B** | **$19.5 B** |
| ***By Income Tier*** | | | | | | | | |
| **LI** | 84,739,921 (11%) | $0.2 B (3) | $0.3 B (3) | $0.6 B (2) | $0.6 B (3) | $0.9 B (3) | $0.9 B (5) | $0.9 B (5) |
| **LMI1** | 230,936,378 (30%) | $0.7 B (11) | $1.4 B (12) | $2.6 B (11) | $2.6 B (12) | $4.3 B (15) | $4.3 B (22) | $4.2 B (22) |
| **LMI2** | 94,895,600 (12%) | $0.7 B (12) | $1.4 B (12) | $4.1 B (17) | $4.0 B (20) | $3.8 B (13) | $3.4 B (18) | $3.4 B (18) |
| **UMI1** | 263,137,970 (35%) | $2.8 B (46) | $5.3 B (45) | $10.6 B (43) | $10.1 B (49) | $9.0 B (31) | $7.5 B (38) | $7.5 B (38) |
| **UMI2** | 86,992,729 (11%) | $1.7 B (28) | $3.4 B (29) | $6.5 B (26) | $3.5 B (16) | $10.1 B (36) | $3.5 B (18) | $3.5 B (18) |
| ***By World Region*** | | | | | | | | |
| **EAP** | 295,355,455 (39%) | $2.6 B (42) | $4.6 B (39) | $10.2 B (42) | $10.2 B (497) | $8.1 B (29) | $8.1 B (41) | $8.0 B (41) |
| **ECA** | 39,367,836 (5%) | $0.5 B (9) | $1.0 B (9) | $2.0 B (8) | $1.7 B (8) | $2.5 B (9) | $1.7 B (8) | $1.7 B (9) |
| **LAC** | 82,566,777 (11%) | $1.7 B (29) | $3.5 B (30) | $6.7 B (27) | $3.3 B (16) | $10.8 B (38) | $3.2 B (16) | $3.2 B (16) |
| **MENA** | 27,105,360 (4%) | $0.2 B (3) | $0.4 B (4) | $1.1 B (4) | $1.1 B (5) | $0.9 B (3) | $0.8 B (4) | $0.8 B (4) |
| **SA** | 220,923,612 (29%) | $0.6 B (10) | $1.2 B (10) | $2.3 B (10) | $2.3 B (11) | $3.7 B (13) | $3.7 B (19) | $3.6 B (19) |
| **SSA** | 95,083,558 (13%) | $0.5 B (8) | $1.0 B (9) | $2.1 B (9) | $2.1 B (10) | $2.1 B (8) | $2.1 B (11) | $2.2 B (11) |
| ***10-year roll-out***^b^**, *cost (% of total costs)*** | | | | | | | | |
| **TOTAL** | 760,402,598 | **$4.2 B** | **$7.9 B** | **$16.5 B** | **$14.0 B** | **$19.1 B** | **$13.3 B** | **$13.2 B** |
| ***By Income Tier*** | | | | | | | | |
| **LI** | 84,739,921 (11%) | $0.1 B (3) | $0.2 B (3) | $0.4 B (2) | $0.4 B (3) | $0.6 B (3) | $0.6 B (5) | $0.6 B (5) |
| **LMI1** | 230,936,378 (30%) | $0.5 B (11) | $0.9 B (12) | $1.8 B (11) | $1.8 B (13) | $2.9 B (15) | $2.9 B (22) | $2.9 B (22) |
| **LMI2** | 94,895,600 (12%) | $0.5 B (12) | $0.9 B (12) | $2.8 B (17) | $2.7 B (20) | $2.6 B (13) | $2.3 B (18) | $2.3 B (18) |
| **UMI1** | 263,137,970 (35%) | $1.9 B (46) | $3.5 B (45) | $7.2 B (43) | $6.8 B (49) | $6.1 B (31) | $5.0 B (38) | $5.0 B (38) |
| **UMI2** | 86,992,729 (11%) | $1.2 B (27) | $2.3 B (6) | $4.4 B (26) | $2.3 B (16) | $6.9 B (36) | $2.3 B (18) | $2.3 B (18) |
| ***By World Region*** | | | | | | | | |
| **EAP** | 295,355,455 (39%) | $1.8 B (42) | $3.1 B (39) | $6.9 B (42) | $6.9 B (49) | $5.4 B (29) | $5.4 B (41) | $5.4 B (41) |
| **ECA** | 39,367,836 (5%) | $0.4 B (9) | $0.7 B (9) | $1.4 B (8) | $1.2 B (8) | $1.7 B (9) | $1.1 B (8) | $1.1 B (9) |
| **LAC** | 82,566,777 (11%) | $1.2 B (28) | $2.4 B (30) | $4.5 B (27) | $2.2 B (16) | $7.3 B (38) | $2.2 B (16) | $2.2 B (16) |
| **MENA** | 27,105,360 (4%) | $0.1 B (3) | $0.3 B (4) | $0.7 B (4) | $0.7 B (5) | $0.6 B (3) | $0.6 B (4) | $0.6 B (4) |
| **SA** | 220,923,612 (29%) | $0.4 B (10) | $0.8 B (10) | $1.6 B (10) | $1.6 B (11) | $2.5 B (13) | $2.5 B (19) | $2.5 B (19) |
| **SSA** | 95,083,558 (13%) | $0.3 B (8) | $0.7 B (9) | $1.4 B (9) | $1.4 B (10) | $1.5 B (8) | $1.5 B (11) | $1.5 B (11) |

^a^ LMIC: low- and middle-income countries; LI: Low Income; LMI1: Lower-middle income tier 1; LMI2: Lower-middle income tier 2; UMI1: Upper-middle income tier 1; UMI2: Upper-middle income tier 2; EAP: East Asia & Pacific; ECA: Europe & Central Asia; LAC: Latin America & Caribbean; MENA: Middle East & North Africa; SA: South Asia; SSA: Sub-Saharan Africa.

^b^ Immediate roll-out: Full coverage (100%) of the target population from 2015 to 2024; 5-year roll-out: 20% coverage in 2015, 40% coverage in 2016, 60% coverage in 2017, 80% coverage in 2018, and 100% coverage of the target population from 2019 to 2024; 10-year roll-out: 10% coverage in 2015, 20% coverage in 2016, 30% coverage in 2017, 40% coverage in 2018, 50% coverage in 2019, 60% coverage in 2020, 70% coverage in 2021, 80% coverage in 2022, 90% coverage in 2023, and 100% coverage of the target population in 2024.

**Figure A. Unit costs by procedure in included countries, relative to GNI per capita (2013 US$).**

**References**

1. Ferlay J, Soerjomataram I, Ervik M, Dikshit R, Eser S, Mathers C, et al. (2013) GLOBOCAN 2012 v1.0, Cancer Incidence and Mortality Worldwide: IARC CancerBase No. 11 [Internet]. Lyon, France: International Agency for Research on Cancer.

2. Jeronimo J, Bansil P, Lim J, Peck R, Paul P, Amador JJ, et al. A multicountry evaluation of careHPV testing, visual inspection with acetic acid, and papanicolaou testing for the detection of cervical cancer. Int J Gynecol Cancer 2014; 24: 576-585.

3. Arbyn M, Sankaranarayanan R, Muwonge R, Keita N, Dolo A, Mbalawa CG, et al. Pooled analysis of the accuracy of five cervical cancer screening tests assessed in eleven studies in Africa and India. Int J Cancer 2008; 123: 153-160.

4. Arbyn M, Verdoodt F, Snijders PJ, Verhoef VM, Suonio E, Dillner L, et al. Accuracy of human papillomavirus testing on self-collected versus clinician-collected samples: a meta-analysis. Lancet Oncol 2014; 15: 172-183.

5. Qiao YL, Sellors JW, Eder PS, Bao YP, Lim JM, Zhao FH, et al. A new HPV-DNA test for cervical-cancer screening in developing regions: a cross-sectional study of clinical accuracy in rural China. Lancet Oncol 2008; 9: 929-936.

6. Institut Catala d'Oncologia. ICO Information Centre on HPV and Cancer. Available at: www.hpvcentre.net. Accessed on September 9, 2014.

7. Guan P, Howell-Jones R, Li N, Bruni L, de Sanjose S, Franceschi S, et al. Human papillomavirus types in 115,789 HPV-positive women: a meta-analysis from cervical infection to cancer. Int J Cancer 2012; 131: 2349-2359.

8. Zhang R, Velicer C, Chen W, Liaw KL, Wu EQ, Liu B, et al. Human papillomavirus genotype distribution in cervical intraepithelial neoplasia grades 1 or worse among 4215 Chinese women in a population-based study. Cancer Epidemiol 2013; 37: 939-945.

9. Muwonge R, Wesley RS, Nene BM, Shastri SS, Jayant K, Malvi SG, et al. Evaluation of cytology and visual triage of human papillomavirus-positive women in cervical cancer prevention in India. Int J Cancer 2014; 134: 2902-2909.

10. Qiao YL, Jeronimo J, Zhao FH, Schweizer J, Chen W, Valdez M, et al. Lower cost strategies for triage of human papillomavirus DNA-positive women. Int J Cancer 2014; 134: 2891-2901.

11. Hutubessy R, Levin A, Wang S, Morgan W, Ally M, John T, et al. A case study using the United Republic of Tanzania: costing nationwide HPV vaccine delivery using the WHO Cervical Cancer Prevention and Control Costing Tool. BMC Med 2012; 10: 136.

12. Levin CE, Van Minh H, Odaga J, Rout SS, Ngoc DN, Menezes L, et al. Delivery cost of human papillomavirus vaccination of young adolescent girls in Peru, Uganda and Viet Nam. Bull World Health Organ 2013; 91: 585-592.

13. Quentin W, Terris-Prestholt F, Changalucha J, Soteli S, Edmunds WJ, Hutubessy R, et al. Costs of delivering human papillomavirus vaccination to schoolgirls in Mwanza Region, Tanzania. BMC Med 2012; 10: 137.

14. Campos NG, Maza M, Alfaro K, Gage JC, Castle PE, Felix JC, et al. The comparative and cost-effectiveness of HPV-based cervical cancer screening algorithms in El Salvador. Int J Cancer 2015.

15. Programme for Research and Capacity Building in Sexual and Reproductive Health and HIV in Developing Countries. Costs of cervical cancer screening in Ghana. London: UK Department for International Development, 2010.

16. Goldie SJ, Gaffikin L, Goldhaber-Fiebert JD, Gordillo-Tobar A, Levin C, Mahe C, et al. Cost-effectiveness of cervical-cancer screening in five developing countries. N Engl J Med 2005; 353: 2158-2168.

17. Mvundura M, Tsu V Estimating the costs of cervical cancer screening in high-burden Sub-Saharan African countries. Int J Gynaecol Obstet 2014; 126: 151-155.

18. World Bank. World Development Indicators. Available at: http://data.worldbank.org/data-catalog/world-development-indicators. Accessed on September 9, 2014.
